# Reduced adaptation of glutamatergic stress response is associated with pessimistic expectations in depression

**DOI:** 10.1038/s41467-021-23284-9

**Published:** 2021-05-26

**Authors:** Jessica A. Cooper, Makiah R. Nuutinen, Victoria M. Lawlor, Brittany A. M. DeVries, Elyssa M. Barrick, Shabnam Hossein, Daniel J. Cole, Chelsea V. Leonard, Evan C. Hahn, Andrew P. Teer, Grant S. Shields, George M. Slavich, Dost Ongur, J. Eric Jensen, Fei Du, Diego A. Pizzagalli, Michael T. Treadway

**Affiliations:** 1 Department of Psychology, Emory University, Atlanta, GA USA; 2 Center for Depression, Anxiety and Stress Research, McLean Hospital/Harvard Medical School, Belmont, MA USA; 3 Department of Psychiatry and Behavioral Sciences, Emory University, Atlanta, GA USA; 4 Department of Psychological Science, University of Arkansas, Fayetteville, AR USA; 5 Cousins Center for Psychoneuroimmunology and Department of Psychiatry and Biobehavioral Sciences, University of California, Los Angeles, CA USA; 6 Department of Psychiatry, McLean Hospital/Harvard Medical School, Belmont, MA USA; 7 McLean Imaging Center, McLean Hospital/Harvard Medical School, Belmont, MA USA

**Keywords:** Cognitive neuroscience, Stress and resilience

## Abstract

Stress is a significant risk factor for the development of major depressive disorder (MDD), yet the underlying mechanisms remain unclear. Preclinically, adaptive and maladaptive stress-induced changes in glutamatergic function have been observed in the medial prefrontal cortex (mPFC). Here, we examine stress-induced changes in human mPFC glutamate using magnetic resonance spectroscopy (MRS) in two healthy control samples and a third sample of unmedicated participants with MDD who completed the Maastricht acute stress task, and one sample of healthy control participants who completed a no-stress control manipulation. In healthy controls, we find that the magnitude of mPFC glutamate response to the acute stressor decreases as individual levels of perceived stress increase. This adaptative glutamate response is absent in individuals with MDD and is associated with pessimistic expectations during a 1-month follow-up period. Together, this work shows evidence for glutamatergic adaptation to stress that is significantly disrupted in MDD.

## Abstract (web summary)

Stress is a major risk for mental illness that is known to impact glutamate function in the medial prefrontal cortex (mPFC). Using magnetic resonance spectroscopy we find evidence for an adaptive mPFC glutamate response to stress in healthy adults that is notably impaired in patients with major depression.

## Introduction

Stress is a major risk factor for physical and psychological health problems^1^ and has been strongly linked to the onset of major depressive disorder (MDD)^1,2^. Although ‘stress’ is often broadly defined, prior research has divided this construct into ‘good stress’, ‘tolerable stress’, and ‘toxic stress’, with the latter being associated with significant risk for physiological damage and mental illness^3^. Toxic stressors are frequently characterized by a lack of predictability and controllability^4^ and are often related to social threat, such as isolation, rejection, and exclusion^5,6^. One of the most widely replicated consequences of toxic stressors is stress-induced anhedonia, resulting in behavioral inhibition and a failure to pursue rewards^7–9^. Stress reduces acquisition of reward-related information^8,10^ and blunts activity in corticostriatal regions involved in reward processing, including the medial prefrontal cortex (mPFC), dorsal striatum, and orbitofrontal cortex^11,12^. Importantly, responses to stress are sensitive to individual differences, with diminished reward sensitivity only being observed in stress-reactive individuals^13^. Additionally, elevated perceptions of stress have been found to confer particular risk for blunted reward processing^4,8,14^, with self-reported levels of perceived stress predicting blunted neural responses to monetary reward in the mPFC^14^. To date, however, the neural mechanisms of stress-induced anhedonia and interactive effects of acute and perceived stress remain unclear.

The medial prefrontal cortex (mPFC) has emerged as a critical region that may underlie stress-induced anhedonia. A robust preclinical literature has elucidated numerous negative effects of stress in mPFC, including glutamate-mediated excitotoxicity that may result from frequent elevations of circulating glucocorticoids^15,16^. In the rodent mPFC, for example, initial stress exposure has been shown to increase extracellular glutamate^17^, potentiate post-synaptic excitatory currents^18^, and upregulate surface expression of glutamate alpha-amino-3-hydroxy-5-methyl-4-isoxazolepropionic acid (AMPA) and *N*-methyl-*D*-Aspartate (NMDA) receptors^19^. These effects have, in turn, been linked to adaptive changes, including short-term enhancements in learning and memory. With repeated stress exposure, however, glutamate release in response to subsequent acute stressors shows rapid habituation^17^. Similarly, animals previously exposed to chronic stress demonstrate reduced potentiation of glutamatergic signaling when faced with a subsequent stressor^20^. This reduction in mPFC glutamate in response to stress and concomitant reductions in dendritic arbors and spines in mPFC^21,22^ have been proposed as possible protective mechanisms that facilitate a necessary adaptation to repeated toxic stressors^3^.

Localization of the above effects to the mPFC is particularly relevant for understanding how perceived stress may lead to the development of stress-related psychopathology. Substantial work has consistently implicated overlapping roles for the mPFC in coordinating behavioral and endocrine responses to stress^23,24^ as well as the valuation of expected rewards^25,26^. The mPFC in particular plays critical roles in representing the expectations and probabilities for future outcomes^27^. Additionally, animal studies have strongly implicated this region in both risk and resilience for learned helplessness behavior, where individuals form expectations that their actions are incapable of impacting future outcomes^28^. Taken together, this literature suggests that repeated stress exposure may significantly alter mPFC glutamate function, which in turn may contribute to depressive phenotypes. A critical unanswered question, however, is the extent to which the attenuated mPFC glutamate responses to new stressors represents a protective adaptation or a negative consequence, given individual perceptions of recent stress.

Here, we examine changes in glutamate following an acute stressor and how these changes relate to perceived stress using magnetic resonance spectroscopy (MRS), a method that has been widely used to examine in vivo changes in glutamatergic metabolites^29–31^. We first examine glutamate metabolites in a sample of healthy adults with varying levels of recent perceived stress before and after an acute stressor that was designed to be unpredictable, minimally controllable, and include a social threat component. We hypothesized that for healthy individuals with low levels of perceived stress, mPFC glutamate would increase following an acute stressor, whereas for individuals with higher levels of perceived stress, mPFC glutamate would decrease. We then replicate this experiment in a second sample of healthy adults. To determine the specificity of these effects to acute stressor (as opposed to mere exposure to any cognitive task), we evaluate a third sample of healthy adults using a “no stress” control manipulation designed to mimic the sensory and cognitive components of the acute stressor. Next, we examined the relationship between acute stress-induced mPFC glutamate changes and recent perceived stress in a sample of participants with MDD, hypothesizing that adaptive mPFC glutamate stress responses would be disrupted in MDD. Consistent with our hypotheses, we find that in healthy control participants, but not participants with depression, the magnitude of mPFC glutamate response to the acute stress task decreases as individual levels of perceived stress increase. Finally, to understand how this disrupted response might relate to anhedonia, we evaluate associations between mPFC glutamate function and reward processing in daily life using ecological momentary assessment (EMA). We find that the lack of an adaptive glutamate response predicts pessimistic expectations in daily life. Together, this work shows evidence for glutamatergic adaptation to stress that is significantly disrupted in MDD.

## Results

### Effects of the acute stress manipulation on mood and salivary cortisol

**Fig. 1:**
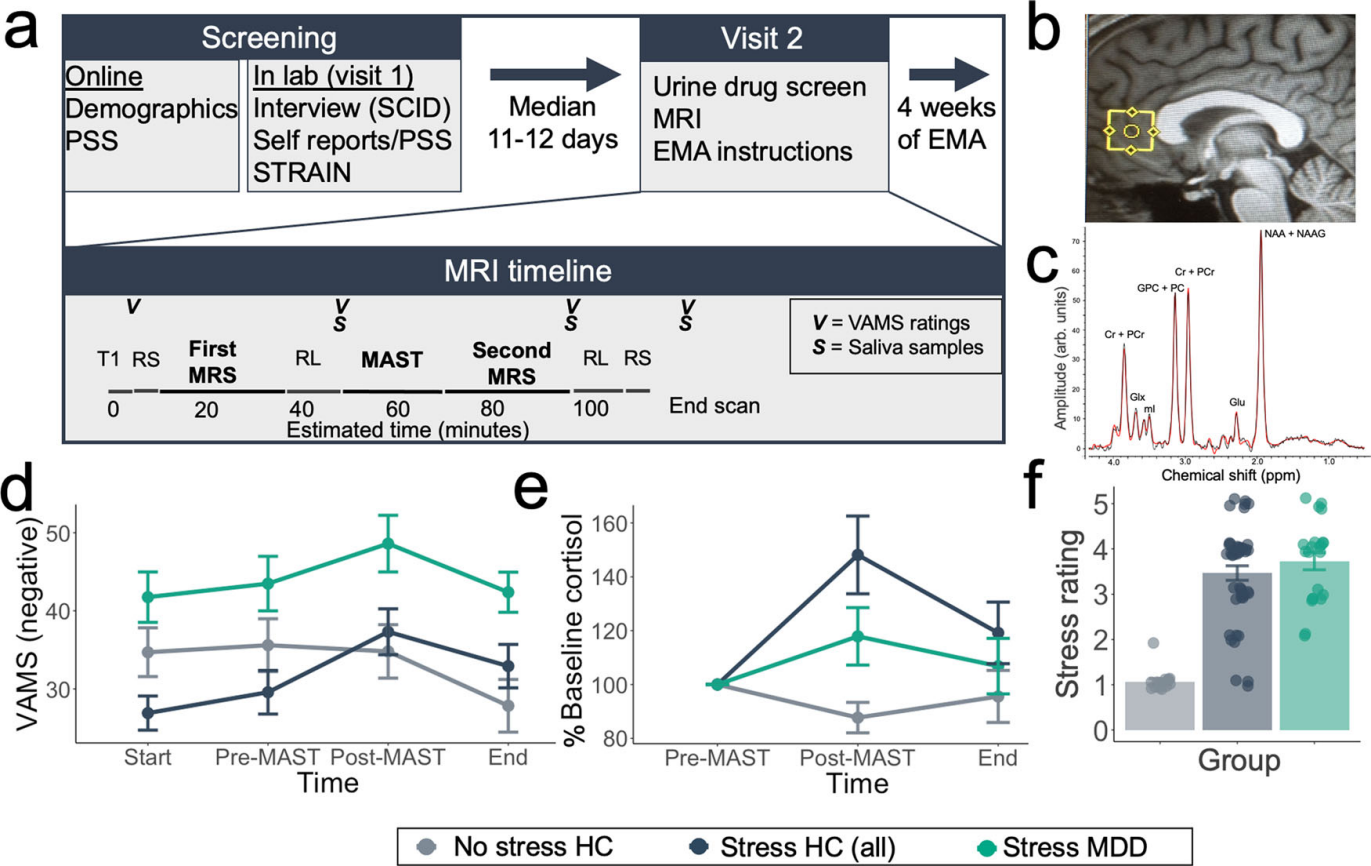
Study design and effects of stress on salivary cortisol and subjective ratings. **a** Schematic diagram of the study visits and approximate timing of MRS, fMRI RL task, VAMS, and saliva measurements. Note that the Healthy Control Stress sample did not complete resting-state scans, STRAIN, or EMA. EMA Ecological Momentary Assessment, MRS Magnetic Resonance Spectroscopy, PSS Perceived Stress Scale, RL Reinforcement Learning, RS Resting State, SCID Structured Clinical Interview for DSM Disorders, STRAIN Stress and Adversity Inventory. MAST Maastricht Acute Stress Test, VAMS Visual Analog Mood Scales. **b** Representative MRS voxel placement. **c** Representative MRS spectrum (black) and LCModel fit (red) with labeled metabolite peaks. arb.units arbitrary units, Cr creatine, PCr Phosphocreatine, Glu glutamate, Glx (glutamine + glutamate), GCP glycerophosphocholine and PC, phosphocholine (choline-containing metabolites), ml myo-inositol, MRS Magnetic Resonance Spectroscopy, NAA N-acetylaspartic acid, NAAG N-acetylaspartylglutamate, ppm parts per million. **d** Effect of MAST acute stress task and No Stress Control (NSC) on mood. Items are coded such that higher scores indicate greater negative emotional experience and averaged across items. Data represented as mean ± standard error of the mean (*N* = 75 participants). **e** Salivary cortisol response to acute stress manipulation and no-stress control. Graph depicts percent change in salivary cortisol from the timepoint immediately prior to the onset of the MAST stressor (Pre-MAST). Data represented as mean ± standard error of the mean (*N* = 83 participants). **f** Subjective stress ratings for each group (1–5). Data represented as mean ± standard error of the mean (*N* = 82 participants). HC Healthy Control participants, MDD participants with Major Depressive Disorder. Source data for **d**–**f** are provided as a Source Data file.

Participants in this study included healthy controls (HC) across three independent samples and a fourth sample of unmedicated patients meeting criteria for current Major Depressive Disorder (MDD; see Table 1 for demographic information). After completing interview and self-report measures, all participants completed an MRI scanning session that included two MRS assessments of mPFC metabolites on a 3 Tesla (3 T) scanner using a well-validated MRS protocol with excellent test-retest reliability (see Supplementary Information and Fig. 1a-c). In between the first and second MRS scans, participants in two of the healthy control groups and the MDD group completed an acute stress manipulation (Maastricht Acute Stress Task; MAST^32^), whereas the third sample of healthy control participants completed a No-Stress Control (NSC) manipulation. To confirm the success of our stress manipulation, we first examined changes in mood using an adapted version of the visual analogue mood scale (VAMS^33^; see Methods). Participants were included in a 4 (Timepoint) × 3 (Group) repeated-measures ANOVA if they had complete VAMS data from all four timepoints (*N* = 75). Group included No Stress Control (NSC), Healthy Control Stress (combined samples), and participants with major depressive disorder (MDD). We found a significant main effect of Timepoint (*F*_(2.5, 182.55)_ = 8.71, *p* < 0.001), and main effect of Group (*F*_(2,72)_ = 5.43, *p* = 0.006), as well as a significant Timepoint × Group interaction (*F*_(5.07,182.55)_ = 3.95, *p* = .002). Among participants who completed the acute stress manipulation, we observed a significant effect of Timepoint (*F*_(2.40, 138.90)_ = 11.95, *p* < .001) and main effect of Diagnostic Group (*F*_(1,58)_ = 9.70, *p* = .003), but no significant Timepoint × Diagnostic Group interaction (*F*_(2.40 138.90)_ = 1.34, *p* = 0.265), indicating that the MDD and control groups exhibited similar decreases in mood following the acute stressor, whereas participants with MDD reported higher negative emotional experience overall (Fig. 1d). We additionally compared healthy control participants who completed the stressor vs no-stress control. While the main effect of Acute Stress was not significant (*F*_(1, 52)_ = 0.12, *p* = 0.726), we observed as significant Timepoint × Acute Stress interaction (*F*_(2.46,127.72)_ = 8.10, *p* < 0.001). Whereas healthy control participants who completed the stress manipulation showed peak negative emotional experience following the MAST stressor, negative affect for the NSC group was consistent throughout the scan and lowest at the end of the study (Fig. 1d).

**Table 1:** Demographics and self-report measures.

	Healthy control stress (*n* = 25)	Healthy control stress replication (*n* = 22)	No-stress control (*n* = 18)	Participants with major depressive disorder stress (*n* = 23)
Sex (% Female)	60.00%	68.80%	77.80%	69.60%
Age	26.04 ± 6.20	28.36 ± 8.21	23.44 ± 4.40	29.87 ± 10.61
PSS	10.12 ± 3.70	9.00 ± 5.04	12.11 ± 5.45	27.43 ± 5.89

*PSS* Perceived Stress Scale.

In addition to mood effects, we examined changes in salivary cortisol, which is a widely used marker of the stress response (Fig. 1e). We compared cortisol values taken from immediately prior to the onset of the acute stressor (and after habituation to the scanner environment) to the two poststressor timepoints collected approximately 20- and 40-min poststressor. All timepoints were scaled relative to the prestress timepoint to represent percent change in cortisol from baseline and included in a repeated-measures ANOVA. Participants were only included in this analysis if they had sufficient cortisol from all three timepoints (*N* = 83). We observed a marginally significant effect of Timepoint (*F*_(1.62,129.94)_ = 3.03, *p* = 0.063), marginally significant main effect of Group (*F*_(2,80)_ = 2.86, *p* = 0.063), and significant Timepoint × Group interaction (*F*_(3.25,129.94)_ = 3.19, *p* = 0.023). Among participants who completed the stress manipulation (healthy controls and participants with MDD), we observed a main effect of Timepoint (*F*_(1.57, 98.66)_ = 6.94, *p* = 0.003) and significant quadratic effect of Timepoint (*F*_(1,63)_ = 11.49, *p* = 0.001); conversely, the Timepoint × Diagnostic Group interaction (*F*_(1.57, 98.66)_ = 1.45, *p* = 0.239) and the main effect of Diagnostic Group (*F*_(1,63)_ = 1.31, *p* = 0.256) were not significant. The magnitude of the cortisol effect (*d* = 0.37) was consistent with the average cortisol effect size from stress studies reported in Dickerson & Kemeny (2004)^5^ (*d* = 0.31; see Supplementary Information). Among healthy control participants, we observed a significant Timepoint × Acute Stress interaction (*F*_(1.54,90.95)_ = 5.28, *p* = 0.012) and a significant Timepoint × Acute Stress quadratic contrast (*F*_(1,59)_ = 9.05, *p* = 0.004). Whereas cortisol increased relative to baseline for healthy controls at the first timepoint following the stress manipulation (*t*_42_ = 3.33, *p* = 0.002), participants in the NSC group showed a slight decrease in cortisol concentration following the no-stress control manipulation (*t*_17_ = −2.18, *p* = 0.044).

Finally, we examined participants’ subjective ratings collected at the end of the scan (*N* = 82), which included their subjective levels of stress, unpleasantness, and difficulty of the water/counting manipulation (Fig. 1f), using ANOVA, with Group (HC stress, NSC, and MDD stress) as a between-subjects factor. Main effects of Group were highly significant for all three questions (*ps* < 1.0 × 10^−13^), driven by lower ratings of the NSC group. For participants who completed the stress manipulation, no significant effects of Diagnostic Group were observed for subjective levels of stress, unpleasantness, or difficulty (*p*s > 0.18).

### Effects of perceived stress on mPFC glutamate following acute stress manipulation in healthy control participants

**Fig. 2:**
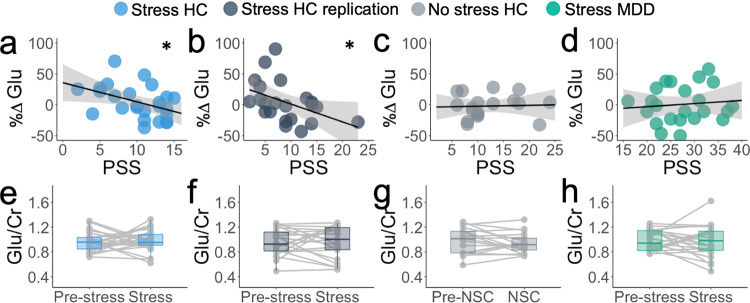
Changes in mPFC creatine-normalized glutamate in response to acute and perceived stress. **a** Association between perceived stress (PSS scores) and percent change in Glu/Cr signal (*r*_s(23)_ = −0.457, *p* = 0.022, two-tailed, uncorrected) in healthy control stress sample. **b** Association between perceived stress (PSS scores) and percent change in Glu/Cr signal (*r*_s(20)_ = −0.517, *p* = 0.014, two-tailed, uncorrected) in the healthy control stress replication sample. **c** Association between perceived stress (PSS scores) and percent change in Glu/Cr signal (*r*_s(16)_ = 0.139, *p* = 0.581, two-tailed, uncorrected) in no-stress control sample. **d** Association between perceived stress (PSS scores) and percent change in Glu/Cr signal (*r*_s(21)_ = 0.115, *p* = 0.602, two-tailed, uncorrected) in participants with major depressive disorder. Shaded area on **a**–**d** represents 95% confidence interval, *p < 0.05. **e**–**h** Glu/Cr ratios before and after MAST in **e** healthy control stress sample (*n* = 25 participants), **f** healthy control stress replication (*n* = 22 participants), **g** no-stress control (*n* = 18 participants), and **h** participants with major depression (*n* = 23 participants). Boxplot elements for e-h indicate median (center line), first and third quartiles (box limits; 25–75th percentile), smallest observation within 1.5 times the interquartile range from the lower quartile (bottom whisker), largest observation within 1.5 times the interquartile range from the upper quartile (top whisker), and all individual participants (points). Cr Creatine-containing metabolites (Creatine and Phosphocreatine), Glu glutamate, HC healthy control, MAST Maastricht Acute Stress Test, MDD participants with major depressive disorder, NSC no-stress control, PSS Perceived Stress Scale. Source data are provided as a Source Data file.

Having established the validity of our acute stress and NSC manipulations, we next sought to test our primary hypothesis regarding the effects of acute stress on mPFC glutamate in the first Healthy Control Stress sample (*n* = 25; McLean Hospital sample). We hypothesized that recent perceived stress as measured by the Perceived Stress Scale (PSS^34^) would predict changes in mPFC glutamate under stress such that healthy individuals with low PSS scores would show greater mPFC glutamate following the acute stress manipulation relative to those with higher PSS scores. Consistent with this hypothesis, percent change in mPFC creatine-normalized glutamate (%ΔGlu; Eq. (1)) was inversely associated with participants’ PSS scores (*r*_s_ = −0.457, *p* = 0.022). Individuals with low PSS scores exhibited an increase in mPFC glutamate following acute stress, whereas individuals with higher PSS scores showed either no change or a slight decrease in mPFC glutamate levels (Fig. 2a).

To confirm the reproducibility of the relationship between PSS scores and %ΔGlu, we collected a second independent sample of healthy control participants (*n* = 22; Healthy Control Stress Replication; Emory sample). As in our first sample, PSS scores were inversely correlated with %ΔGlu with a similar effect size (*r*_s_ = −0.517, *p* = 0.014; Fig. 2b). Relationships between %ΔGlx and PSS are reported for both groups in Supplementary Fig. 1 and were consistent with correlations observed for %ΔGlu. No main effects of acute stress on Glu/Cr or Glx/Cr were observed for either sample (paired *t p*s > 0.7; Fig. 2e, f, Supplementary Fig. 1e, f), though when examining only individuals reporting low levels of recent perceived stress (PSS scores < 10), the acute stress manipulation did evoke a significant increase in mPFC Glu/Cr (*t*_22_ = 2.39, *p* = 0.026; Supplementary Table 1 and Supplementary Fig. 2). Further, within-subject stability of Glu/Cr was affected by the stress manipulation (see Supplementary Information and Supplementary Fig. 3), suggesting that acute stress may affect mPFC glutamate in different directions across individuals.

Next, we sought to determine whether the association between PSS scores and %ΔGlu following the acute stress manipulation was specific to the acute stress manipulation as compared to the No Stress Controls (NSC) condition. Participants in the NSC condition showed no association between %ΔGlu and PSS scores (*r*_s_ = 0.139, *p* = 0.581; Fig. 2c). To confirm that the association between PSS scores and %ΔGlu was significantly stronger during the acute stress manipulation relative to the NSC condition, we additionally examined the interaction between the acute stress manipulation and PSS using hierarchical linear regression. PSS Score and dummy-coded Acute Stress condition were entered in the first block, whereas Study Site, Age, Sex, and the PSS × Acute Stress condition interaction term were entered in the second block using stepwise selection. The PSS × Acute Stress interaction term and Age were both significant predictors of %ΔGlu. The PSS × Acute Stress condition interaction term was associated with decreased %ΔGlu ($\beta$ = −0.40, *t*_60_ = −2.15, *p* = 0.035), while Age was associated with increased %ΔGlu ($\beta$ = 0.29, *t*_60_ = 2.43, *p* = 0.018). No other variables were significant predictors of %ΔGlu (*p*s > 0.4; see Supplementary Table 2). This model explained a significant proportion of the variance in %ΔGlu (adjusted *R*^2^ = 0.196*; F*_(4,60)_ = 4.91, *p* = 0.002), and the change in *R*² from including the PSS × Acute Stress interaction was significant (ΔR² *F-change*_(1,60)_ = 4.63, *p* = 0.035). The model was also run controlling for Cramér–Rao lower bound (CRBL) of glutamate and with %ΔGlx, revealing a similar pattern of results (see Supplementary Tables 2-3).

**Fig. 3:**
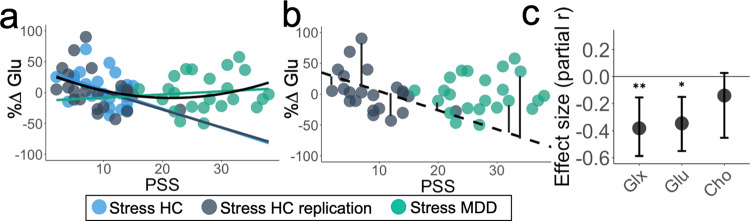
Relationship between perceived stress and MRS metabolites. **a** Relationship between PSS and change in creatine-normalized glutamate in all participants who completed the acute stress manipulation. Group-level linear trends (colors) and combined quadratic effect (black) are overlaid. **b** Maladaptive glutamate response was calculated for the healthy control stress replication sample (gray) and participants with major depressive disorder (green), defined as the residual between the observed percent change glutamate and expected percent change glutamate, estimated using the linear function of the healthy control stress sample, shown in black. Percent change glutamate expected = 35.647 − 3.093*PSS. **c** Partial effect size (Pearson’s *r*; controlling for age and sex, two-tailed, uncorrected) between PSS and percent change glx (glutamate and glutamine; *r*_partial(43)_ = −0.382, *p* = 0.010), percent change glutamate (*r*_partial(43)_ = −0.346, *p* = 0.020), and percent change choline-containing metabolites (*r*_partial(43)_ = −0.141, *p* = 0.356) in all healthy controls who completed the acute stress manipulation (*n* = 47 participants). Significance indicated as *p < 0.05, **p < 0.01. Estimates and error bars (95%CI) were estimated using bootstrapping with 1000 samples. Cho Choline-containing metabolites (primarily glycerophosphocholine and phosphocholine), Glu Glutamate, Glx Glutamate + Glutamine, HC Healthy Control, MDD Participants with major depressive disorder, MRS Magnetic Resonance Spectroscopy, PSS Perceived Stress Scale. Source data are provided as a Source Data file.

Finally, to test whether the effects described above were attributable to a global association with PSS across metabolites, we ran the same regression model to predict percent change in choline-containing metabolites (primarily glycerophosphocholine and phosphocholine; %ΔCho). This model did not explain a significant portion of variance in %ΔCho (adjusted *R*^2^ = 0.011*, F*_(2,62)_ = 1.34, *p* = 0.27) (See Fig. 3c for PSS-metabolite effect sizes and Supplementary Table 4).

### Effects of objective stress on mPFC glutamate following acute stress manipulation in healthy control participants

While the PSS measures how unpredictable and uncontrollable respondents find their life (i.e., stress appraisal), it does not yield an objective assessment of stressors experienced by each participant. To provide a more objective characterization of stress exposure, we used the recently-developed, computer-adapted Stress and Adversity Inventory (STRAIN^35^), though we note that the STRAIN was not available at the time of data collection for the McLean sample. An advantage of the STRAIN is that it provides a more objective quantification of the number of all stressors experienced, and can therefore help determine whether associations between PSS and %ΔGlu observed in the healthy control sample were more likely driven by stress exposure or perceptions of stress. Interestingly, we found no significant associations between %ΔGlu and STRAIN assessments of count or severity of either acute life events and chronic difficulties (*r*_*s*_ values ranged from −0.148 to 0.102*, ps* > 0.5). We additionally examined associations between %ΔGlu and STRAIN assessments of count and severity of acute life events and chronic difficulties experienced only within the last year, again finding no significant associations (*r*_*s*_ values ranged from 0.008 to 0.089, *p*s > 0.7).

Taken together, these results suggest that perceived stress reliably predicts changes in mPFC glutamate following acute stress in healthy individuals, and that our observed associations with PSS may be related to overall subjective appraisal of recent stress. Moreover, the fact that all participants in these samples had no history of psychiatric illness despite moderate levels of PSS suggests that the observed decrease in mPFC glutamate as PSS scores increased may reflect a beneficial adaptation.

### Effects of acute and perceived stress on glutamate in major depressive disorder (MDD)

To further understand how perceived stress may drive mPFC glutamate responses to an acute stressor, we next evaluated a sample of participants with current MDD, a disorder strongly linked to stress exposure and significant elevations in PSS scores^36^. In contrast to our healthy control samples, PSS scores and %ΔGlu following the acute stress manipulation were not significantly correlated in participants with MDD (*r*_s_ = 0.115, *p* = 0.602; Fig. 2d). We used hierarchical linear regression across all three samples that completed the acute stress manipulation, with PSS Scores and dummy-coded Diagnostic Group (HC or MDD) entered in the first block and Age, Sex, Study Site, and the PSS × Diagnostic Group interaction term entered in the second block using stepwise selection. Only PSS ($\beta$ = −0.97, *t*_66_ = −3.10, *p* = 0.003) and the PSS × Diagnostic Group interaction term ($\beta$ = 0.77, *t*_66_ = 2.48, *p* = 0.016) were significant predictors of %ΔGlu (Fig. 3a, Supplementary Table 5). This model explained a significant proportion of the variance in %ΔGlu (adjusted *R*^2^ = 0.093, *F*_(3,66)_ = 3.36, *p* = 0.024) and the change in *R*² from including the PSS × Diagnostic Group interaction was significant (ΔR² *F-*change_(1,66)_ = 6.15, *p* = 0.016). The model was also run controlling for the CRLB of glutamate and with %ΔGlx and revealed a similar pattern of results (Supplementary Tables 5-6).

Although both samples of healthy control participants showed a negative relationship between PSS and %ΔGlu, PSS and %ΔGlu were not significantly correlated in participants with MDD. Additionally, without considering diagnostic status, the relationship between PSS and %ΔGlu following the acute stressor was predicted by a model that included a quadratic PSS term (PSS-squared) (adjusted *R*^2^ = 0.077, *F*_(2,67)_ = 3.88, *p* = 0.025; Fig. 3a, Supplementary Tables 7-8). Relative to the linear function (i.e., only including PSS), the introduction of the quadratic PSS term explained an additional 7.8% of the variance in %ΔGlu (ΔR² *F-*change_(1,67)_ = 5.86, *p* = 0.018).

### Main effects of depression on mPFC glutamate

The main effect of depression on glutamate signal and interactions with acute stress (Fig. 2e-h) were examined using a repeated-measures ANOVA with levels of glutamate (Glu/Cr) at each Timepoint (pre- and poststress) as a within-subjects factor and Diagnostic Group (MDD/control) as a between-subject factor. The main effect of Diagnostic Group (*F*_(1,68)_ = 0.197, *p* = 0.658), Timepoint (*F*_(1,68)_ = 0.001, *p* = .975) and Timepoint × Diagnostic Group interaction (*F*_(1,68)_ = 0.150, *p* = 0.699) were all nonsignificant (see Supplementary Tables 9-12 for metabolite values). This comparison was also conducted using Glx/Cr, finding consistent results (*p*s > 0.9*)*. Glu/Cr and Glx/Cr ratios at baseline showed no differences between participants with MDD and healthy controls (*p*s > 0.4), nor were Glu/Cr and Glx/Cr ratios different between the diagnostic groups after being exposed to the acute stress manipulation (*p*s > 0.9). These results suggest that mPFC glutamate at baseline and in response to acute stress did not differ between healthy control participants and participants with MDD. Associations with between Glu/Cr and age were also examined (see Supplementary Information). Across all participants, Glu/Cr at baseline was negatively correlated with age, *r*_86_ = −0.237, *p* = 0.026 (Supplementary Fig. 4).

### mPFC glutamate response and experience of reward in daily life

Next, we sought to determine how altered mPFC glutamate responses might be related to expectations about events in daily life. Because the interpretation of %ΔGlu depends on PSS, we developed a “maladaptive glutamate response” (MGR) metric that represented the difference between the actual %ΔGlu and the level that would be expected given a participant’s rating of recent perceived stress (Fig. 3b, Eq. (2)). To avoid any non-independence in this analysis, the slope used to calculate the MGR in Eq. (2) was defined by only the McLean sample. We then tested whether the MGR was related to the expectations, experienced outcomes, or affective ratings in daily life collected over a 4-week follow-up period using ecological momentary assessment (EMA) in the Emory samples. Our EMA protocol was designed with particular emphasis on assessing the accuracy and inaccuracy of expectations for daily activities. Expectation inaccuracy was quantified as the difference between the experienced outcome for an activity and the outcome that the participant anticipated experiencing, similar to a reward prediction error under reinforcement learning frameworks. Descriptions of each EMA variable are included in Table 2.

**Table 2:** Ecological momentary assessment (EMA) variable descriptions.

EMA variable	Survey items	Calculation
Expected outcome	Choose an activity from the list below that you will probably do in the NEXT 2 H. Rate your expectation for this event	For all surveys: mean of expectations
Experienced outcome	Last time you chose an event from the activity/event list that you thought might happen. Did it happen? How was it?	For activities that happened: mean of experienced outcomes
Expectation inaccuracy	Rate your expectation for this event and How was it?	For all activities that happened: mean of |Outcome–Expectation|
Optimistic expectations	Rate your expectation for this event and How was it?	For all activities where expectations were better than experienced outcomes: mean of (Outcome–Expectation)
Pessimistic expectations	Rate your expectation for this event and How was it?	For all activities where expectations were worse than experienced outcomes: mean of (Outcome–Expectation)
Positive affect	RIGHT NOW how much do you feel each of the following? Enthusiastic, Cheerful, Relaxed	For all survey responses: mean of positive affect items
Negative affect	RIGHT NOW how much do you feel each of the following? Irritable, Anxious, Sad	For all survey responses: mean of negative affect items

See Methods for survey inclusion criteria.

**Fig. 4:**
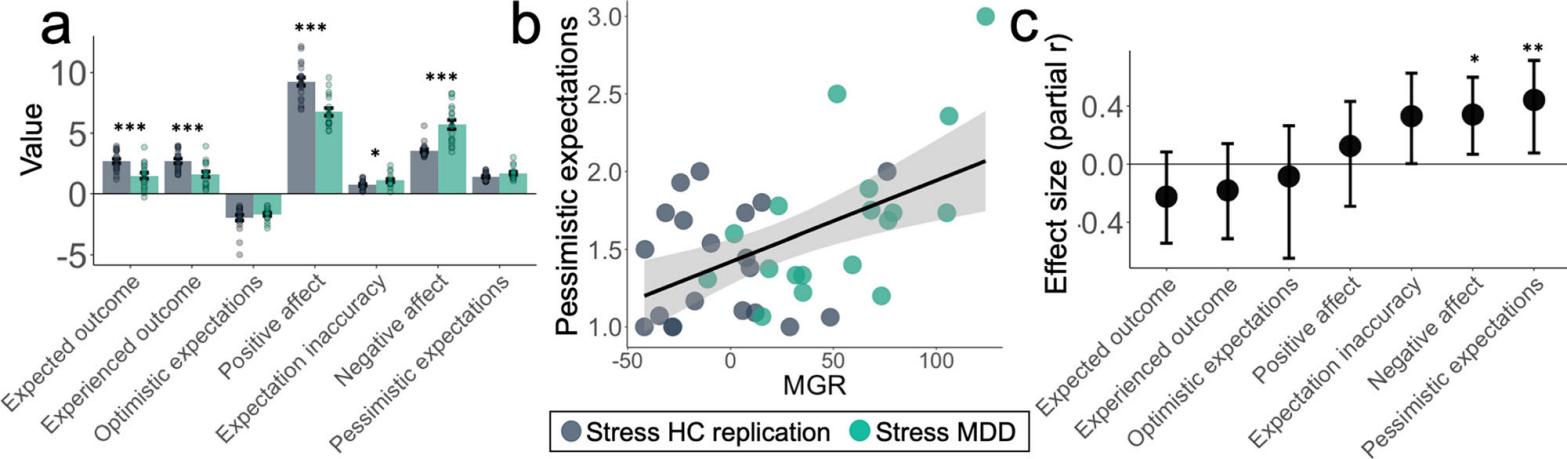
Ecological momentary assessment and associations with maladaptive glutamate response. **a** Differences in ecological momentary assessment (EMA) ratings between healthy control (HC; *n* = 20 participants) and participants with major depressive disorder (MDD; *n* = 18 participants) with significance indicated as *p < 0.05, ***p < 0.001 *(*t-test, two-tailed, uncorrected). Statistical comparisons were *t*_36_ = −3.98, *p* < 0.001 for expected outcomes, *t*_36_ = −3.74, *p* = 0.001 for experienced outcomes, *t*_35_ = 0.96 *p* = 0.342 for optimistic expectations, *t*_36_ = −5.44, *p* < 0.001 for positive affect, *t*_36_ = 2.45, *p* = 0.019 for expectation inaccuracy, *t*_36_ = 5.62, *p* < 0.001 for negative affect, and *t*_36_ = 1.89, *p* = 0.066 for pessimistic expectations. Data represented as mean ± standard error of the mean. **b** Association between maladaptive glutamate response (MGR) and pessimistic expectations from EMA, *r*_36_ = 0.515, *p* < 0.001 (two-tailed, uncorrected). Shaded band represents 95% confidence interval. **c** Effect sizes (partial Pearson’s *r*, two-tailed, uncorrected) between MGR and EMA variables, controlling for age, sex, and diagnostic group (*n* = 38 participants), *r*_partial(33)_ = −0.225, *p* = 0.193 for expected outcomes, *r*_partial(33)_ = −0.181, *p* = 0.299 for experienced outcomes, *r*_partial(32)_ = −0.085, *p* = 0.631 for optimistic expectations, *r*_partial(33)_ = 0.123, *p* = 0.483 for positive affect, *r*_partial(33)_ = 0.330, *p* = 0.053 for ex*p*ectation inaccuracy, *r*_partial(33)_ = 0.341, *p* = 0.045 for negative affect and *r*_partial(33)_ = 0.441, *p* = 0.008 for pessimistic expectations. Effect sizes and error bars (95%CI) were estimated using bootstrapping with 1000 samples and estimated separately for variables with different numbers of observations. Note that the optimistic expectations comparisons in **a** and **c** had one less degree of freedom than other variables, as one participant had no surveys with optimistic expectations. Source data are provided as a Source Data file.

Compared to healthy controls, participants with MDD reported higher average negative affect (*t*_36_ = 5.62, *p* < 0.001), lower positive affect (*t*_36_ = −5.44, *p* < 0.001), lower expected outcomes for activities (*t*_36_ = −3.98, *p* < 0.001), and lower experienced outcomes for activities (*t*_36_ = −3.74, *p* = 0.001). On average, participants with depression had a lower proportion of responses with accurate expectations (M = 0.38) than healthy control participants (M = 0.53; *t*_36_ = −2.35, *p* = 0.024; see Supplementary Fig. 5 for distributions). The average magnitude of expectation inaccuracy was also greater in participants with depression (*t*_36_ = 2.45, *p* = 0.019), indicating less accurate estimations of outcomes (Fig. 4a). We examined directionality of expectation inaccuracies by calculating the mean inaccuracy when expectations were lower than the experienced outcome (“pessimistic expectations”) and when expectations were higher than the experienced outcome (“optimistic expectations”). Participants with depression had a higher proportion of responses with pessimistic expectations (*M* = 0.38) than healthy control participants (*M* = 0.28; *t*_36_ = 2.11, *p* = 0.042; Supplementary Fig. 5), and marginally higher proportion of events with optimistic expectations (*M* = .24) than healthy control participants (*M* = 0.19; *t*_36_ = 1.71, *p* = 0.095). Participants with MDD had marginally higher magnitude of inaccuracies from pessimistic expectations (*t*_36_ = 1.89, *p* = 0.066), but did not differ in mean inaccuracies from optimistic expectations (*p* = 0.34), suggesting that participants with MDD experienced pessimistic expectations more often than healthy controls, and that their pessimistic expectations were slightly more negative than those of healthy controls.

Maladaptive glutamate response (MGR) was compared to EMA variables, controlling for age, sex, and diagnostic group with pairwise exclusions. MGR was positively associated with negative affect (*r*-partial = 0.367, *p* = 0.030), as well as pessimistic expectations (*r*-partial = 0.457, *p* = 0.006; Fig. 4b), but not optimistic expectations (*r*-partial = −0.096, *p* = 0.590; see Fig. 4c). When additionally controlling for participants’ PSS scores, the association between MGR and pessimistic expectations remained significant (*r*-partial = 0.353, *p* = 0.040), whereas the relation between MGR and negative affect did not reach significance (*r*-partial = 0.281, *p* = 0.107). The relationship between MGR and magnitude (mean) of pessimistic expectations remained significant when additionally controlling for frequency of pessimistic expectations (*r*-partial = 0.387, *p* = 0.026). Among participants in which both optimistic and pessimistic expectations were observed (*n* = 37) we additionally compared the partial correlations, controlling for age, sex, and diagnostic group, between MGR and pessimistic expectations and MGR and optimistic expectations using Steiger’s-Z^37,38^ and found that the relationship between MRG and pessimistic expectations (*r*-partial = 0.473) was stronger than the relationship between MGR and optimistic expectations (*r*-partial = −0.085; Z = 2.11, *p* = 0.035).

## Discussion

In this study, we characterized an adaptive glutamatergic response to acute stress in the human mPFC. In two independent samples, we found that healthy individuals exhibited a clear reduction of mPFC glutamate response to a new stressor (%ΔGlu) as their levels of recent perceived stress increased. Critically, this effect was absent for unmedicated individuals with current MDD, suggesting that this absence of adaptation may be a contributor to stress-related psychiatric disease. Further, the extent to which individuals failed to exhibit an attenuated mPFC %ΔGlu response was linked to negative functioning in daily life.

The observed relationship between perceived stress and mPFC Glu suggests an important adaptation to stress among healthy control participants. Importantly, participants from these two samples were confirmed to have no history of psychiatric illness, despite the fact that their perceived stress levels extended into the moderate range^39^. This suggests that some of our participants exhibited resiliency in the face of mild-to-moderate perceived stressors, supporting the notion that attenuated glutamate response may represent an appropriate adaptation to an elevated allostatic load. Under models of allostatic regulation, biological and behavioral responses to an acute stressor should be influenced by levels of recent perceived stress^3,15^. Consistent with this framework, preclinical studies have provided clear evidence that glutamate transmission is potentiated by acute stress and stress hormone exposure^16,18,19^, and that this effect is reversed if an acute stressor is experienced in the context of recent stress^15,17,20^. Critically, attenuation of the glutamate response among individuals with high perceived stress was attributable to the experience of an acute stressor; when healthy control participants completed a task that matched the cognitive and sensory components of the MAST but was reported as non-stressful (see Fig. 1d-f), there was no association between perceived stress and %ΔGlu.

To further determine whether the association between glutamate and perceived stress was adaptive, we next examined the response to acute stress in participants with MDD, a population known to be associated with excessive stress exposure and impaired coping. Participants with MDD reported significantly higher levels of perceived stress relative to healthy control participants. However, these elevated PSS scores did not show the same association with mPFC %ΔGlu following acute stress that was observed in two independent healthy control samples. Indeed, moderation analysis confirmed a significant difference in how PSS scores predicted mPFC %ΔGlu response to acute stress as a function of MDD.

Although participants with MDD as a whole did not show the same inverse association between perceived stress and %ΔGlu, we observed significant variability in glutamate change following acute stress. Using the slope from one of the healthy control samples as an independent quantitative estimate for appropriate %ΔGlu given a certain level of perceived stress, we were able to quantify a “maladaptive %ΔGlu response” (MGR) and examine whether this metric was associated with expectations for activities in daily life. During our 4-week EMA follow-up period, we found that the MGR predicted a consistent pattern of inaccurately low expectations for future events—when activities went better than expected, high MGR was associated with reduced accuracy (i.e., activities were expected to be much less positive than they actually were). This effect remained strong even while controlling for depression diagnosis, PSS score, and frequency of pessimistic expectations. It is further notable that this effect was only evident for occasions when outcomes were better than expected, suggesting it may play a critical role in stress-induced anticipatory anhedonia. Moreover, our results underscore the critical role for anticipation and expectation setting in the clinical phenomenology of anhedonia^4,40^. Interestingly, this aspect of anticipatory anhedonia could also be construed as being part of the general tendency of depressed patients to make overly negative predictions about future events^41,42^. Indeed, a much broader preclinical and human neuroimaging literature has repeatedly implicated mPFC as a region involved in estimating the expected value for future options^26,43^ and self-related valuation in general^44^. This raises an important question as to the boundary conditions between excessive pessimism/high negative affect and anhedonia/low positive affect. Further research will be required to determine whether the observed MGR is primarily associated with anhedonia, negative affect, or both.

It is also notable that while a replicable association emerged between mPFC %ΔGlu and the PSS, we did not observe any association between mPFC %ΔGlu and various indices of stress exposure as indexed by the STRAIN. One potential reason for this lack of effect is a difference in timescales between the STRAIN and PSS; while the PSS focuses on appraisal of stressful experiences over the last month only, the STRAIN assesses lifetime stress exposure. That said, we did not observe any significant associations between the STRAIN and glutamate even when limiting measures of stress exposure to the previous year, suggesting that mPFC %ΔGlu in response to stress may be related to stress exposure over even shorter timescales. Alternatively, this dissociation could be attributable to fundamentally different components of stress captured by the PSS and STRAIN. Whereas the STRAIN objectively quantifies the number of moderate-to-major life stressors experienced, the PSS assesses subjective feelings of uncontrollability, unpredictability, and generally feeling “stressed.” This appraisal may be more akin to chronic low-level stressors, feelings of being “stressed out”, and inability to cope that contribute to allostatic load^3,45^. Both explanations are plausible and they are not mutually exclusive. We also note that we were only able to collect the STRAIN in the Emory samples, which may have limited our ability to detect associations. We also wish to highlight that the salivary cortisol response to stress was not associated with %ΔGlu, as preclinical studies have suggested that glucocorticoids play a critical role in shaping prefrontal glutamate responses to stress^16^. These findings are consistent with previous work^46^ and may be attributable to limitations in the temporal resolution of our MRS and saliva measurements. Future research will be needed to determine how adaptation of mPFC %ΔGlu is related to the perception and timescale of stressful experiences.

Collectively, these findings have a number of implications for our conceptualization of biological adaptations to stress and their potential role in psychiatric disorders. Our study reveals that recent perceived stress reliably moderates mPFC glutamate responses to a novel acute stressor in psychiatrically healthy individuals, but not in those experiencing depression. This is notable, as it suggests that the effects of mPFC glutamate levels depend critically on context. Although glutamate dysfunction has been implicated in MDD^15,47^, we did not observe depression-related differences in basal glutamate, Glx, or glutamatergic response to acute stress, suggesting that cross-sectional comparisons of resting metabolites alone may be insufficient to serve as a reliable biomarker. These findings are consistent with a recent meta-analysis of MRS studies that found no evidence for basal differences in mPFC glutamate associated with MDD and no significant difference in Glx in unmedicated patients with MDD^48^. In addition to limiting the generalizability of our findings, the inclusion of only unmedicated participants may have limited our ability to detect differences in glutamate metabolites at baseline. We also note that basal differences in glutamatergic metabolites have been shown to be related to anhedonic symptoms^49^ and number of depressive episodes^50^. Effects of medication, anhedonia, and number of depressive episodes should be explored in relation to glutamatergic response to stress in future work.

The present study is not without limitations. Our hypotheses regarding stress and mPFC glutamate were primarily informed on the basis of preclinical studies that were able to measure synaptic glutamate and post-synaptic excitatory currents, while MRS glutamate signal is primarily driven by intracellular glutamate and cannot be used to make direct inferences about glutamate transmission or synaptic release. Despite this limitation, prior fMRS studies and meta-analyses suggest that pain or stressful stimuli can induce reliable changes in MRS metabolites that are consistent with expected changes based on preclinical studies^29,31,51^. Our acute stress manipulation did evoke a significant increase in glutamate for healthy individuals with low levels of recent perceived stress. Further, the acute stress manipulation affected the variance of glutamate. Consistent with previous work using MRS^46^, within-subject levels of glutamate were correlated for participants in the no-stress control condition, but not for participants who completed the stress manipulation, suggesting important variability in glutamatergic response to stress across individuals. Thus, while the precise interpretation of changes in glutamate may be unclear, it can still serve as a potential biomarker for individual differences in response to stress.

We acknowledge additional limitations related to our sample size and range of age and PSS. Our samples were only of moderate size, which was partly due to the exclusion of participants with poor-quality MRS data. To address this concern, we recruited a replication sample of healthy controls, and found very similar effect sizes for the relationship between the PSS and %ΔGlu. We were also unable to recruit healthy control participants and participants with MDD with fully overlapping distributions of PSS scores, despite a robust pre-screen effort using online recruitment tools. This was not entirely unexpected, as PSS scores are known to be much higher in MDD samples^36^; however, it does limit our ability to determine whether the maladaptive glutamate response we observed was driven primarily by the high severity of perceived stress in MDD, the presence of their current depression, or both. Finally, we observed that increasing age was positively associated with %ΔGlu, possibly suggesting increased glutamatergic stress reactivity. However, our sample included a limited age range and thus cannot be extrapolated to patients with MDD in adolescence or older adulthood. We note that previous work in participants older than those included in our study has found that stress reactivity, as measured by cortisol response, is reduced in older adults^52^. We additionally found that baseline glutamate levels in mPFC were negatively correlated with age, consistent with previous work^53^. Although glutamate levels are thought to be steady in childhood and late adolescence^54^, little is known about glutamatergic response to stress in adolescence and the age range of our sample limits our ability to make inferences about age-related effects on glutamatergic stress reactivity in adolescence.

In sum, this study is the first that we know of to identify attenuation of mPFC %ΔGlu as an adaptive response to acute stress in the context of perceived stress, and to demonstrate how this response is impaired in individuals with depression. These results advance our understanding of the neurobiological adaptation to stress, and may play a valuable role in identifying new treatment targets and markers of treatment response in human stress-related illness.

## Methods

### Participants

Adults (age 18–60) participated in this study across three independent samples of healthy controls (HC) and a fourth sample of unmedicated patients meeting criteria for current major depressive disorder (MDD). The first sample of healthy control participants (Healthy Control Stress) was recruited at the McLean Imaging Center (McLean Hospital) in response to community advertisements in Boston, MA, whereas the three replication, extension, and patient samples (i.e., Healthy Control Stress Replication, No Stress Control, Major Depressive Disorder Stress) were recruited at the Facility for Education and Research in Neuroscience (FERN) neuroimaging center at Emory University in Atlanta, GA. To ensure a range of perceived stress scores, individuals recruited for the three samples collected at Emory first completed an online eligibility screening in REDCap^55^ that included the Perceived Stress Scale (PSS^34^) and additional demographic and eligibility questions.

### Eligibility criteria

For healthy control participants in all samples, participants were excluded for any current or past psychiatric disorder, with the exception of specific phobia, or past alcohol abuse, as assessed by the Structured Clinical Interview for the DSM-IV (SCID)^56^ administered by a trained master’s level clinician. For participants in the MDD group, diagnosis of MDD was confirmed using the SCID. Additional exclusion criteria for participants with MDD included current substance abuse or dependence, obsessive-compulsive disorder, bipolar disorder, active suicidal ideation as assessed by the Columbia-Suicide Severity Rating Scale (C-SSRS^57^), or any form of psychotic disorder. Participants with MDD with comorbid anxiety disorders or post-traumatic stress disorder were not excluded from the study. Participants in all samples were excluded for recent use of any psychotropic medications or illegal drugs, which was confirmed using a urine drug screen immediately prior to scanning. Exclusion criteria also included current use or more than occasional use in the past year of tobacco products, as assessed by subject report.

In total, 124 participants met inclusion criteria and participated in the MRI visit (*n*_control_ = 93, *n*_MDD_ = 31). Thirteen participants did not finish the scan visit due to time constraints, undiagnosed claustrophobia, subject illness, inability to fit comfortably in the scanner, or scanner malfunction. Exclusion criteria for MRS data included signal to noise ratio (SNR) less than 9, full width at half maximum (FWHM) greater than 0.15, Cramér-Rao Lower Bound (CRLB) for glutamate greater than 20%, and poor spectral quality based on visual inspection. Quality of MRS data from the McLean sample was reviewed by JEJ, while quality of MRS data from the Emory samples were reviewed by MTT and FD (MR Physicist, blind to study results), with excellent agreement between ratings, Cohen’s κ = 0.871, *p* < 0.001. In cases of disagreement, judgement was deferred to FD. Twenty-two participants had at least one MRS session of insufficient quality. One additional participant was excluded for a change in glutamate over three standard deviations from the mean, resulting in a final sample size of 88. Only participants from the final sample (“study completers”) were included in subsequent analyses. Sample demographics for study completers in each group are provided in Table 1 and comorbidities for study completers with MDD are included in Supplementary Table 13.

### Study description

All recruitment and testing procedures were approved by the Partners Institutional Review Board (McLean Hospital) and the Emory University Institutional Review Board. During an initial study visit and after informed consent, participants were interviewed using the DSM-IV SCID^56^ to confirm eligibility criteria and completed self-report questionnaires. During the second visit, participants completed an initial MRS scan, a reinforcement learning (RL) task, and an acute stress or no-stress control task (described below), followed by a secondary MRS scan and RL task. Resting-state and task fMRI data were also collected but were not included in these analyses. Salivary cortisol samples were collected before and after the stress (or no stress) manipulation to determine the presence of a stress response (see Fig. 1a and e).

### Acute stress manipulation

To induce stress during the scanning session, participants completed the Maastricht Acute Stress Task^32^. The MAST is a laboratory stress paradigm that combines alternating periods of well-validated stress-inducing procedures, specifically a cold pressor and performance of serial subtraction in front of evaluators. During the cold pressor, participants were instructed to immerse their hand up to and including the wrist into ice water (1–8 °C). Water immersion occurred five times for varying time intervals (30–90s) using a fixed randomized sequence that was unknown to participants so as to create a sense of unpredictability. Between water immersion periods, participants were asked to perform serial subtraction starting from 2043 and counting down by 17; with every mistake, a neutral evaluator instructed the participant to restart from 2043. There were 4 serial subtraction blocks, varying in duration between 30 and 90s. Although the MAST protocol we followed was not originally developed for the scanner environment, all procedures were completed while the participant remained in position in the scanner. The scanner bed was moved out part way to facilitate access of the participant’s hand to a container of cold water. We note that this protocol represented our own MRI-related adaptation of the MAST, and is slightly distinct from the fMRI adaptation developed by Smeets and colleagues (the “iMAST”^58^) though both procedures are highly similar to the original MAST protocol.

### No-stress control manipulation

Participants in the “no stress control” (NSC) condition were instructed to complete a task that followed the same design and timing as the MAST, but used water at a comfortable temperature (26–36 °C) instead of cold water and were asked to count aloud starting from one instead of serial subtraction. Frequency and duration of immersion and counting were determined by computer in the same manner as the MAST. This manipulation was designed to be as similar to the MAST stressor as possible without inducing a stress response.

### Salivary cortisol analysis

Salivary cortisol was collected as indicated in Fig.1a. Samples were stored −20 °C until they were assayed in duplicate for cortisol using a commercially available chemiluminescence immunoassay (CLIA) from IBL-International, Hamburg, Germany (Cortisol Luminescence Immunoassay). Cortisol from saliva samples were assayed at the Laboratory for Biological Health Psychology at Brandeis University (Directors: Dr. Nicolas Rohleder and Dr. Jutta Wolf). Inter- and intra-assay coefficients were below 10%. Changes in salivary cortisol following the MAST are shown in Fig. 1e. The effect size of the cortisol response was compared to effect sizes from published studies^5^ using the standard mean-change statistic (see Supplementary Information for calculation).

### Self-report ratings Questionnaires

To assess perceptions of stress, participants were administered the Perceived Stress Scale (PSS^34^). The PSS is a 10-item questionnaire that asks participants about their perceptions of stress over the past month. Importantly, prior studies have shown that this measure predicts individual differences in mPFC responses to reward information^14^ and reward learning abilities^8^, as well as responses to acute stress^59^. Participants in the replication and extension groups also completed the Stress and Adversity Inventory for Adults (STRAIN^35^). The STRAIN is an online stress assessment interview that measures cumulative lifetime exposure to different types of stress that have been shown to predict numerous health-related outcomes, including self-reported mental and physical health problems^60^ and biological reactivity to acute stress^61^. Variables extracted from the STRAIN included the STRAIN’s two main stressor exposure outcomes (i.e., lifetime stressor count and severity) and indices indicating the specific types of stressors experienced (i.e., count and severity of both acute life events and chronic difficulties).

To measure affective responses to the acute stress paradigm (described below), all participants completed mood ratings using an adapted version of the visual analogue mood scale (VAMS^33^). This scale presents participants with five horizontal lines, each representing a bipolar dimensional mood state: Happy-Sad, Relaxed-Tense, Friendly-Hostile, Sociable-Withdrawn, Quick Witted-Mentally Slow. Participants were instructed to move a cursor on each line to the point that best described their current mood state. This VAMS scale was administered before and after the MAST acute stress manipulation (see Fig. 1a). All VAMS ratings were then scaled so that higher scores indicated greater negative emotional experience and averaged for each subject to represent negative emotional experience for each timepoint. Changes in VAMS average ratings for study completers are shown in Fig. 1d. Following the completion of the MRI scan, participants were asked to rate the stress (or no stress) manipulation on difficulty, stress (Fig. 1f), and unpleasantness on a scale from 1 (Not at All) to 5 (Extremely).

### MRS acquisition

For both the McLean and Emory sites, MRS data were collected on a 3 T Siemens Tim TRIO using a 32-channel phased-array design RF head coil operating at 123 MHz for proton imaging and spectroscopy using an identical Proton MRS sequence developed by JEJ. High-resolution T1-weighted anatomical images were used to position a single 2 × 2 × 2 cm^3^ voxel in the mPFC. The voxel was adjusted in the transverse plane as needed for each subject such that the posterior edge of the voxel was placed directly in front of the anterior edge of the corpus callosum and positioned as shown in Fig. 1b. Proton MRS employed a modified J-resolved PRESS protocol (2D-JPRESS), which collects PRESS MRS spectra at incremental echo-times (TE) to sample the J-resolved periodicity of coupled metabolites (e.g., Glu and Gln) for better spectral resolution^62,63^. Shimming of the magnetic field within the prescribed voxel was done automatically using an automated shimming routine followed by a manual shim to further minimize unsuppressed water linewidth and optimize voxel field homogeneity. Following the additional automated optimization of water suppression power, carrier-frequency, tip angles and coil tuning, the 2D-JPRESS sequence collected 22 echo-time (TE)-stepped spectra with the echo-time ranging from 30 ms to 350 ms in 15 ms increments. Acquisition parameters were: repetition time (TR) = 2 s, f1 acquisition bandwidth = 67 Hz, spectral bandwidth = 2 kHz, readout duration = 512 ms, NEX = 16/TE-step, total scan duration = 12 min. The identical sequence was performed twice (Pre-MAST, Post-MAST).

### Test-retest reliability of MRS sequence

The test-retest reliability for J-resolved MRS scans using this protocol at McLean Hospital in an overlapping rostral anterior cingulate cortex ROI has been previously established, with less than 10% variance for Glutamate/Creatine (Glu/Cr) ratios^64^ and intraclass correlation coefficient (ICC) of 0.803^65^. To confirm that we were able to achieve a comparable level of test-retest reliability at the Emory scanning site, six additional participants completed two consecutive MRS scans using our 2D-JPRESS protocol. Consistent with prior work^65^, ICC values for Glu/Cr metabolites were calculated in SPSS v27 (IBM, Armonk, NY) using two‐way mixed models with absolute agreement, finding excellent test-retest reliability (ICC = 0.89, *p* = 0.017; Supplementary Fig. 6).

### MRS analysis

jMRUI 5.2^66^ was used to visually inspect files and to convert data from DICOM to ASCII format for analysis using LCModel version 6.3–1K^67,68^. Spectroscopic data processing and analyses in LCModel were performed on a Linux workstation. To quantify glutamate (Glu) with the JPRESS data, the 22 TE-stepped free-induction decay (FIDs) were first zero-filled out to 64 points (TE-stepped dimension), Gaussian-filtered, and Fourier transformed. Consistent with validated methods^63^, every J-resolved spectral extraction within a bandwidth of 67 Hz was fitted with LCModel and its theoretically-correct template, which used an optimized GAMMA-simulated J-resolved basis sets modeled for 2.89 T (the actual field strengths of Siemens Tim Trio scanners)^63^. The integrated area under the entire 2D surface for each metabolite was calculated by summing the raw peak areas across all 64 J-resolved extractions for each metabolite. Glu metabolites were expressed as ratios to total creatine-containing metabolites (Creatine and Phosphocreatine, included in Eq. (1) as “Cr”). A representative spectrum and associated LC model fit is shown in Fig. 1c. %ΔGlu was calculated using Eq. (1):

1 $$\% \triangle {{\rm{Glu}}}=\frac{{{{\rm{Glu}}}/{{\rm{Cr}}}}_{{{\rm{poststress}}}}-{{{\rm{Glu}}}/{{\rm{Cr}}}}_{{{\rm{prestress}}}}}{{{{\rm{Glu}}}/{{\rm{Cr}}}}_{{{\rm{prestress}}}}}$$

Percent change in Glx (Glutamate and Glutamine) and percent change in choline-containing metabolites (primarily glycerophosphocholine + phosphocholine) were also calculated using Eq. (1). Creatine ratios for each sample of participants are included in Supplementary Tables 9-12.

### Ecological momentary assessment (EMA)

Participants in the replication and extension samples were invited to participate in a 4-week ecological momentary assessment (EMA) protocol to assess reward expectation and experience in daily life. EMA data were collected using Qualtrics survey software (Qualtrics XM; Qualtrics, Provo, UT), with survey links sent to participants’ phones via scheduled text messages. Surveys were sent every other day for a period of four weeks. On active survey days, participants received six surveys spaced by 2 h. Participants were asked to rate their current affect, indicate their planned activity in the next 2 h, indicate whether or not their last planned activity occurred (to provide ratings regarding the outcome of completed activities), and to rate their expected affect. Ratings of current and future affect were collected on a 5-point scale from 1 (“not at all”) to 5 (“extremely”) for positive affect items (enthusiastic, cheerful, and relaxed) and negative affect items (anxious, sad, irritable). Expectations for activities and experienced outcomes were rated on a 9-point scale from −4 (very negative) to +4 (very positive). For the full question and survey flow, see Supplementary Fig. 7. Forty participants completed the EMA protocol (HC: 21, MDD: 19). Two participants were excluded from analysis for having less than 20 usable survey data points, resulting in data from 20 healthy controls and 18 individuals with MDD. The overall survey completion rate was 84.4%, with healthy controls completing 83.9% of surveys and participants with depression completing 85.0% of surveys. Surveys were excluded if they were incomplete, extended beyond the sixth survey of the day, were completed in less than 30 s or more than 24 h, or were not completed within 1 to 3 h following the prior survey, resulting in usable data from 1236 surveys from healthy control participants and 1138 surveys from participants with MDD. Inaccuracy of reward estimation was quantified as the difference between the experienced reward for an activity and the amount of reward that the participant anticipated experiencing (see Table 2), similar to a reward prediction error under reinforcement learning frameworks, and included surveys after the first survey of the day (HC = 813, MDD = 659).

### Maladaptive glutamate response to stress

Adaptive glutamate response under stress was characterized using the linear function between recent perceived stress (PSS) and %ΔGlu in the Healthy Control Stress sample. This out-of-sample linear function was used to estimate the expected %ΔGlu (%ΔGlu_exp_) for each participant from the replication sample and sample of participants with MDD. Maladaptive glutamate response (MGR) was estimated as the difference between observed glutamate change under stress (%ΔGlu_obs_) and %ΔGlu_exp_ (Eq. (2)), where positive values indicate that mPFC glutamate increased more than expected given the participant’s recent perceived stress, and negative values indicate that change in glutamate was less than expected given recent perceived stress.

2 $${{\rm{MGR}}}= \% \Delta {{{\rm{Glu}}}}_{{{\rm{obs}}}}- \% \Delta {{{\rm{Glu}}}}_{\exp }$$

### Statistical analysis

Change in self-report ratings, task performance, and salivary cortisol were analyzed using separate repeated-measures ANOVAs. For cases that violated the sphericity assumption, a Greenhouse-Geisser correction was used. For single sample correlations, Spearman correlations were used to control for possible violations of parametric assumptions that can occur in modest sample sizes and are indicated as r_s_. Interactions between recent perceived stress and the acute stress manipulation in predicting mPFC glutamate levels were examined using hierarchical linear regression, using mean-centered continuous independent variables. Analyses were performed using MATLAB 2013B (MathWorks, Natick, MA), SPSS v27 (IBM, Armonk, NY), and R v3.6.0 (R Core Team). EMA data were analyzed using Jupyter Notebooks 4.4.0 in Python 3.7.1^69^. All statistical tests were two-tailed unless otherwise noted.

### Reporting summary

Further information on research design is available in the Nature Research Reporting Summary linked to this article.

### Supplementary information

Supplementary Information

Reporting Summary

### Source data

Source Data

## Data Availability

Source data are provided with this paper. Due to the sensitive nature of the data, we are not able to make raw data files publicly available. Researchers interested in accessing the data for research purposes may contact either corresponding author and we will work with your institution to establish a data sharing agreement as needed while maintaining appropriate human subjects protections. Source data are provided with this paper.

## Code availability

Custom code used in this study (excluding proprietary third-party software such as LCModel) is available for download via GitHub at https://github.com/TReAD-Lab/Cooper_NatComm_2021 and is also deposited at Zenodo (10.5281/zenodo.4696823)^70^.
