# Supplementary material for: Reduced adaptation of glutamatergic stress response is associated with pessimistic expectations in depression
Source: Nat Commun. 2021 May 26;12:3166. doi: 10.1038/s41467-021-23284-9 (PMC8155144; doi:10.1038/s41467-021-23284-9)
Supplement: Supplementary file 1 — Supplementary Information [file 41467_2021_23284_MOESM1_ESM.pdf]

SUPPLEMENTARY INFORMATION FOR:

Reduced adaptation of glutamatergic stress response is associated with pessimistic expectations in depression

Jessica A. Cooper[1*], Makiah R. Nuutinen[1], Victoria M. Lawlor[1], Brittany A. M. DeVries[1], Elyssa M. Barrick[2], Shabnam Hossein[1], Daniel J. Cole[1], Chelsea V. Leonard[1], Emma C. Hahn[1], Andrew, P. Teer[3], Grant S. Shields[4], George M. Slavich[5], Dost Ongur[6], J. Eric Jensen[7], Fei Du[7], Diego A. Pizzagalli[2,6,7], and Michael T. Treadway[1,3*]

[1]Department of Psychology, Emory University, Atlanta, GA, USA.
[2]Center for Depression, Anxiety and Stress Research, McLean Hospital/Harvard Medical School, Belmont, MA, USA.
[3]Department of Psychiatry and Behavioral Sciences, Emory University, Atlanta, GA, USA.
[4]Department of Psychological Science, University of Arkansas, Fayetteville, AR, USA.
[5]Cousins Center for Psychoneuroimmunology and Department of Psychiatry and Biobehavioral Sciences, University of California, Los Angeles, CA, USA.
[6]Department of Psychiatry, McLean Hospital/Harvard Medical School, Belmont, MA, USA.
[7]McLean Imaging Center, McLean Hospital/Harvard Medical School, Belmont MA, USA.

*Correspondence and requests for materials should be addressed to J.A.C. (email: j.cooper@emory.edu) or to M.T.T (email: mtreadway@emory.edu).

Supplementary Tables: 15
Supplementary Figures: 7

Supplementary Notes

Effect size of cortisol response. In order to compare the effect size of the cortisol response (all stress participants) to the effect sizes of the cortisol response observed in a meta-analysis of 208 stress studies, we computed the standard mean-change statistic *d* as reported in Dickerson and Kemeny (2004)[1]. The mean-change statistic was calculated as the difference between the mean cortisol concentration immediately before the stressor onset (M = 6.087 nmol/L) and at the first timepoint following the acute stressor (M = 8.055 nmol/L), divided by the pre-stress standard deviation (SD = 5.388; $[M_{\text{poststressor}} – M_{\text{prestressor}}]/SD_{\text{prestressor}}]$). The effect size of the cortisol response in our study (d = .37) was similar to the average effect size from all stress studies (d = .31) and the average effect size observed for public speaking/verbal interaction tasks (d = 0.39) reported in Dickerson and Kemeny (2004).

Within-Subject Variability in Glutamate. We additionally examined within-subject associations between Glu/Cr at baseline (pre-stress or pre-NSC) and Glu/Cr following the stress or no stress control (NSC) manipulation. Consistent with previous work[2], Glu/Cr was correlated across timepoints (r_{16} = .528, p = .024) for participants who received the NSC manipulation, while Glu/Cr was not significantly correlated for healthy controls who received the stress manipulation (r_{45} = .158, p = .289). This suggests that the acute stress manipulation did have a significant effect on mPFC glutamate by increasing the variability of glutamate following acute stress. Intraclass correlations (ICCs) for Glu/Cr across timepoints were also significant for the NSC condition (ICC = .675, p = .013), but did not reach significance for healthy control participants who received the stress manipulation (ICC = .274, p = .145). The ICCs for metabolites identified in Auerbach & Pizzagalli (2019)[3] as having high test-retest reliability are shown in **Supplementary Fig. 3**.

Demographic effects and effects of birth control on %ΔGlu. In control analyses, we additionally evaluated the putative effects of demographic variables on %ΔGlu and their potential moderation of perceived stress effects in healthy controls who completed the stress manipulation (combined Emory and McLean samples). We did not observe a significant effect of Sex (p = .842), nor did we observe a significant Sex x PSS interaction (p = .355; **Supplementary Table 14**). Change in glutamate was not significantly different between male and female participants (t_{45} = 1.05, p = .30). Increasing age was associated with increased %ΔGlu in

response to stress (β = .273, p = .049), however, we did not observe a significant Age x PSS interaction (p = .41; **Supplementary Table 14**). Among healthy control female participants who completed the stress manipulation, birth control (oral/IUD) was not associated with %ΔGlu under stress (p = .789), and did not moderate effects of PSS on %ΔGlu (p = 0.952; **Supplementary Table 15**).

Associations between glutamate, cortisol, and mood. Relationships between glutamate, cortisol, and mood were examined using Spearman correlations. Among healthy controls who completed the stress manipulation, percent change glutamate was not significantly correlated with cortisol (percent change relative to baseline) at ~20 or ~40 minutes post-stress (*ps* > .16), VAMS response (percentage change from T1 to T3; r_s = -.271, p = .091), or post-scan subjective ratings (*ps* > .2). VAMS response and cortisol response at ~20 minutes and ~40 minutes post-stressor were also not correlated (*p*s > .5). We repeated these comparisons in participants with MDD, finding no significant associations (*p*s > .2).

Associations between age, sex, and glutamate. We additionally examined effects of age and sex on Glu/Cr at baseline and following acute stress. Across all healthy control participants at baseline (stress and NSC), age was negatively correlated with Glu/Cr, r_{63} = -.332, p = .007. The association between age and Glu/Cr was not observed in participants with depression, r_{21} = -.122, p = .578, but was significant when including all participants at baseline, r_{86} = -.237, p=.026 (**Supplementary Fig. 4**). The association between age and basal glutamate is consistent with previous work[4]. Glu/Cr following stress was not correlated in healthy controls who completed the stress manipulation, r_{45} = .191, p = .199. Sex-related differences Glu/Cr were not observed in healthy controls, patients, or the combined sample at baseline (*p*s > .3), or after the acute stressor (*p*s > .3).

Supplementary Table 1: Mean Cr ratios and Cramér-Rao lower bounds (CRLB) for MRS metabolites pre-stressor and post-stressor in healthy control participants with low PSS (PSS < 10)

Aspartate (Asp)								
Pre-stressor				Post-Stressor				Paired t-test p-value
Mean ratio (Cr)	SD ratio (Cr)	Mean CRLB	SD CRLB	Mean ratio (Cr)	SD ratio (Cr)	Mean CRLB	SD CRLB	
.45	.11	10.22	2.97	.45	.12	10.22	2.43	.98
**Choline Containing Metabolites (Primarily Glycerophosphocholine and Phosphocholine; Cho)**								
**Pre-stressor**				**Post-Stressor**				Paired t-test p-value
Mean ratio (Cr)	SD ratio (Cr)	Mean CRLB	SD CRLB	Mean ratio (Cr)	SD ratio (Cr)	Mean CRLB	SD CRLB	
.77	.23	3.96	.77	.90	.29	4.22	.74	.07
**Glutamate (Glu)**								
**Pre-stressor**				**Post-Stressor**				Paired t-test p-value
Mean ratio (Cr)	SD ratio (Cr)	Mean CRLB	SD CRLB	Mean ratio (Cr)	SD ratio (Cr)	Mean CRLB	SD CRLB	
.91	.19	5.83	1.83	1.03	.22	5.35	2.46	.03
**Glutamine (Gln)**								
**Pre-stressor**				**Post-Stressor**				Paired t-test p-value
Mean ratio (Cr)	SD ratio (Cr)	Mean CRLB	SD CRLB	Mean ratio (Cr)	SD ratio (Cr)	Mean CRLB	SD CRLB	
.24	.09	13.17	2.02	.25	.10	13.39	3.12	.81
**Myo-inositol (ml)**								
**Pre-stressor**				**Post-Stressor**				Paired t-test p-value
Mean ratio (Cr)	SD ratio (Cr)	Mean CRLB	SD CRLB	Mean ratio (Cr)	SD ratio (Cr)	Mean CRLB	SD CRLB	
.72	.19	4.83	1.53	.81	.30	4.22	1.28	.16
**N-Acetylaspartic Acid (NAA) + N-acetylaspartylglutamate (NAAG)**								
**Pre-stressor**				**Post-Stressor**				Paired t-test p-value
Mean ratio (Cr)	SD ratio (Cr)	Mean CRLB	SD CRLB	Mean ratio (Cr)	SD ratio (Cr)	Mean CRLB	SD CRLB	
1.07	.19	3.09	1.24	1.09	.27	3.22	1.48	.61
**Glx (Glutamate + Glutamine)**								
**Pre-stressor**				**Post-Stressor**				Paired t-test p-value
Mean ratio (Cr)	SD ratio (Cr)	Mean CRLB	SD CRLB	Mean ratio (Cr)	SD ratio (Cr)	Mean CRLB	SD CRLB	
1.15	.22	-	-	1.29	.25	-	-	.06

Note: All paired t-tests are two-tailed, uncorrected. Cr = Creatine-containing metabolites (creatine and phosphocreatine). MRS = magnetic resonance spectroscopy; PSS = perceived stress scale. Source data are provided as a Source Data file.

Supplementary Table 2: Hierarchical regression models predicting %ΔGlu and %ΔGlx in healthy control samples.

Acute-perceived stress interaction in healthy controls (Glu)

Model 1	**Predictor**	**B**	**SE**	**Beta**	***t***	***p***	**adj-*r2***	***F***	***p***	***r2* change**	***F change***	***F Change p***
Step 1	Constant	1.22	6.20		0.20	0.844	0.08	3.73	0.030	0.11	3.73	0.030
	Acute Stress	1.76	7.35	0.03	0.24	0.812						
	PSS	-1.79	0.69	-0.32	-2.59	0.012						
Step 2	(Age)			0.31	2.47	0.016						
	(PSS x Stress)			-0.42	-2.19	0.033						
	(Sex)			-0.06	-0.50	0.621						
	(Study Site)			-0.04	-0.30	0.764						
Model 2	**Predictor**	**B**	**SE**	**Beta**	***t***	***p***	**adj-*r2***	***F***	***p***	***r2* change**	***F change***	***F Change p***
Step 1	Constant	3.59	6.04		0.60	0.554	0.15	4.72	0.005	0.08	6.08	0.016
	Acute Stress	-1.52	7.19	-0.03	-0.21	0.833						
	PSS	-1.31	0.69	-0.23	-1.89	0.063						
Step 2	Age	1.22	0.49	0.31	2.47	0.016						
	(PSS x Stress)			-0.40	-2.15	0.035						
	(Sex)			-0.08	-0.71	0.482						
	(Study Site)			-0.09	-0.64	0.527						
Model 3	**Predictor**	**B**	**SE**	**Beta**	***t***	***p***	**adj-*r2***	***F***	***p***	***r2* change**	***F change***	***F Change p***
Step 1	Constant	0.16	6.08		0.03	0.980	0.20	4.91	0.002	0.06	4.63	0.035
	Acute Stress	1.21	7.10	0.02	0.17	0.865						
	PSS	0.50	1.08	0.09	0.47	0.642						
Step 2	Age	1.17	0.48	0.29	2.43	0.018						
	PSS x Stress	-2.90	1.35	-0.40	-2.15	0.035						
	(Sex)			-0.01	-0.04	0.970						
	(Study Site)			-0.11	-0.82	0.418						

Acute-perceived stress interaction in healthy controls (Glx)

Model 1	**Predictor**	**B**	**SE**	**Beta**	***t***	***p***	**adj-*r2***	***F***	***p***	***r2* change**	***F change***	***F Change p***
Step 1	Constant	1.10	5.72		0.19	0.848	0.11	5.15	0.009	0.14	5.15	0.009
	Acute Stress	0.92	6.78	0.02	0.14	0.892						
	PSS	-1.97	0.64	-0.37	-3.08	0.003						
Step 2	(Age)			0.25	2.00	0.051						
	(PSS x Stress)			-0.37	-1.95	0.055						
	(Sex)			-0.05	-0.40	0.693						
	(Study Site)			-0.05	-0.40	0.694						

Note. Stress condition (acute stress or no stress control; “acute stress”) and PSS were entered in the first block and all other predictors were included in the second block with stepwise selection. Model coefficients (adjusted r^2, F) are based on included variables, while t and p are provided for excluded variables. Variables excluded for each model are indicated with parenthesis. Models were run independently (two-tailed) and no corrections for multiple comparisons were applied across models. Glu = Glutamate; Glx = Glutamate + Glutamine; SE = Standard Error; PSS = Perceived Stress Scale.

Supplementary Table 3: Hierarchical regression models predicting %ΔGlu and %ΔGlx in healthy control samples controlling for mean Cramér-Rao lower bound (CRLB) of glutamate.

Acute-perceived stress interaction in healthy controls (Glu)

Model 1	Predictor	B	SE	Beta	*t*	*p*	adj-*r2*	*F*	*p*	*r2* change	*F change*	*F Change p*
Step 1	Constant	11.88	14.48		0.82	0.415	0.07	2.69	0.054	0.12	2.69	0.054
	Acute Stress	0.78	7.47	0.01	0.11	0.917						
	PSS	-1.84	0.70	-0.33	-2.64	0.011						
	Mean Glu CR	-1.71	2.09	-0.10	-0.82	0.418						
Step 2	(Age)			0.30	2.45	0.017						
	(PSS x Stress)			-0.41	-2.08	0.042						
	(Sex)			-0.06	-0.46	0.649						
	(Study Site)			-0.06	-0.39	0.697						

Model 2	Predictor	B	SE	Beta	*t*	*p*	adj-*r2*	*F*	*p*	*r2* change	*F change*	*F Change p*
Step 1	Constant	13.82	13.94		0.99	0.325	0.14	3.68	0.010	0.08	5.99	0.017
	Acute Stress	-2.45	7.30	-0.04	-0.34	0.739						
	PSS	-1.36	0.70	-0.24	-1.95	0.056						
	Mean Glu CR	-1.64	2.01	-0.10	-0.82	0.418						
Step 2	Age	1.21	0.50	0.30	2.45	0.017						
	(PSS x Stress)			-0.39	-2.05	0.045						
	(Sex)			-0.08	-0.67	0.508						
	(Study Site)			-0.10	-0.73	0.470						

Model 3	Predictor	B	SE	Beta	*t*	*p*	adj-*r2*	*F*	*p*	*r2* change	*F change*	*F Change p*
Step 1	Constant	7.23	13.96		0.52	0.607	0.19	3.94	0.004	0.05	4.20	0.045
	Acute Stress	0.49	7.26	0.01	0.07	0.946						
	PSS	0.41	1.10	0.07	0.37	0.710						
	Mean Glu CR	-1.12	1.98	-0.07	-0.56	0.575						
Step 2	Age	1.17	0.48	0.29	2.41	0.019						
	PSS x Stress	-2.80	1.37	-0.39	-2.05	0.045						
	(Sex)			0.00	-0.03	0.974						
	(Study Site)			-0.12	-0.87	0.387						

Acute-perceived stress interaction in healthy controls (Glx)

Model 1	Predictor	B	SE	Beta	*t*	*p*	adj-*r2*	*F*	*p*	*r2* change	*F change*	*F Change p*
Step 1	Constant	4.85	13.41		0.36	0.719	0.10	3.42	0.023	0.14	3.42	0.023
	Acute Stress	0.58	6.92	0.01	0.08	0.934						
	PSS	-1.99	0.65	-0.38	-3.08	0.003						
	Mean Glu CR	-0.60	1.94	-0.04	-0.31	0.758						
Step 2	(Age)			0.25	1.98	0.053						
	(PSS x Stress)			-0.37	-1.91	0.060						
	(Sex)			-0.05	-0.38	0.706						
	(Study Site)			-0.06	-0.43	0.669						

Note. Stress condition (acute stress or no stress control; "acute stress"), PSS, and Cramér-Rao lower bound (CRLB) of glutamate (mean Glu CR) were entered in the first block and all other predictors were included in the second block with stepwise selection. Model coefficients (adjusted r^2, *F*) are based on included variables, while *t* and *p* are provided for excluded variables. Variables excluded for each model are indicated with parenthesis. Models were run independently (two-tailed) and no corrections for multiple comparisons were applied across models. Glu = Glutamate; Glx = Glutamate + Glutamine; SE = Standard Error; PSS = Perceived Stress Scale.

Supplementary Table 4: Hierarchical regression models predicting %ΔCholine-containing metabolites (primarily glycerophosphocholine + phosphocholine) in healthy control samples.

Model 1	**Predictor**	**B**	**SE**	**Beta**	***t***	***p***	**adj-*r2***	***F***	***p***	***r2* change**	***F change***	***F Change p***
Step 1	Constant	2.39	7.70		0.31	0.757	0.01	1.34	0.269	0.04	1.34	0.269
	Acute Stress	7.23	9.13	0.10	0.79	0.431						
	PSS	-1.04	0.86	-0.15	-1.21	0.232						
Step 2	(Age)			0.08	0.59	0.559						
	(PSS x Stress)			-0.12	-0.56	0.576						
	(Sex)			-0.01	-0.04	0.968						
	(Study Site)			-0.14	-0.95	0.346						

Note. Stress condition (acute stress or no stress control; “acute stress”) and PSS were entered in the first block and all other predictors were included in the second block with stepwise selection. Model coefficients (adjusted r^2, F) are based on included variables, while t and p are provided for excluded variables. Variables excluded for each model are indicated with parenthesis. All models were run independently (two-tailed) and no corrections for multiple comparisons were applied across models. SE = Standard Error; PSS = Perceived Stress Scale.

Supplementary Table 5: Hierarchical regression models predicting %ΔGlu and %ΔGlx in all stress samples.

Diagnostic Group x PSS interaction in individuals who completed the acute stress manipulation (Glu)

Model 1	**Predictor**	**B**	**SE**	**Beta**	***t***	***p***	**adj-r2**	***F***	***p***	***r2* change**	***F change***	***F Change p***
Step 1	Constant	-3.53	5.94		-0.59	0.555	0.02	1.83	0.168	0.05	1.83	0.168
	Diagnostic Group	20.04	14.71	0.33	1.36	0.178						
	PSS	-1.32	0.71	-0.44	-1.85	0.069						
Step 2	(PSS x Diagnostic Group)			0.77	2.48	0.016						
	(Age)			0.16	1.32	0.191						
	(Sex)			-0.04	-0.36	0.721						
	(Study Site)			-0.03	-0.21	0.837						
Model 2	**Predictor**	**B**	**SE**	**Beta**	***t***	***p***	**adj-r2**	***F***	***p***	***r2* change**	***F change***	***F Change p***
Step 1	Constant	-12.86	6.85		-1.88	0.065	0.09	3.36	0.024	0.08	6.15	0.016
	Diagnostic Group	7.46	15.06	0.12	0.50	0.622						
	PSS	-2.91	0.94	-0.97	-3.10	0.003						
Step 2	PSS x Diagnostic Group	3.42	1.38	0.77	2.48	0.016						
	(Age)			0.11	0.95	0.345						
	(Sex)			0.03	0.24	0.812						
	(Study Site)			-0.06	-0.43	0.666						

Diagnostic Group x PSS interaction in individuals who completed the acute stress manipulation (Glx)

Model 1	**Predictor**	**B**	**SE**	**Beta**	***t***	***p***	**adj-r2**	***F***	***p***	***r2* change**	***F change***	***F Change p***
Step 1	Constant	-3.92	5.55		-0.71	0.483	0.02	1.77	0.179	0.05	1.77	0.179
	Diagnostic Group	20.96	13.74	0.36	1.53	0.132						
	PSS	-1.24	0.67	-0.45	-1.87	0.066						
Step 2	(PSS x Diagnostic Group)			0.86	2.80	0.007						
	(Age)			0.13	1.04	0.301						
	(Sex)			-0.04	-0.36	0.721						
	(Study Site)			-0.04	-0.25	0.807						
Model 2	**Predictor**	**B**	**SE**	**Beta**	***t***	***p***	**adj-r2**	***F***	***p***	***r2* change**	***F change***	***F Change p***
Step 1	Constant	-13.64	6.33		-2.16	0.035	0.11	3.90	0.013	0.10	7.82	0.007
	Diagnostic Group	7.86	13.90	0.14	0.57	0.574						
	PSS	-2.90	0.87	-1.04	-3.34	0.001						
Step 2	PSS x Diagnostic Group	3.55	1.27	0.86	2.80	0.007						
	(Age)			0.07	0.62	0.537						
	(Sex)			0.04	0.31	0.755						
	(Study Site)			-0.07	-0.51	0.614						

Note. Diagnostic group and PSS were entered in the first block and all other predictors were included in the second block with stepwise selection. Model coefficients (adjusted r^2, F) are based on included variables, while t and p are provided for excluded variables. Variables excluded for each model are indicated with parenthesis. Models were run independently (two-tailed) and no corrections for multiple comparisons were applied across models. Glu = Glutamate; Glx = Glutamate + Glutamine; SE = Standard Error; PSS = Perceived Stress Scale.

Supplementary Table 6: Hierarchical regression models predicting %ΔGlu and %ΔGlx in all stress samples controlling for mean Cramér-Rao lower bound (CRLB) of glutamate.

Diagnostic Group x PSS interaction in individuals who completed the acute stress manipulation (Glu)

Model 1	**Predictor**	**B**	**SE**	**Beta**	***t***	***p***	**adj-*r2***	***F***	***p***	***r2* change**	***F change***	***F Change p***
Step 1	Constant	20.97	13.79		1.52	0.133	0.06	2.55	0.063	0.10	2.55	0.063
	Diagnostic Group	21.97	14.44	0.36	1.52	0.133						
	PSS	-1.35	0.70	-0.45	-1.93	0.058						
	Mean Glu CR	-4.33	2.21	-0.23	-1.96	0.054						
Step 2	(PSS x Diagnostic Group)			0.75	2.45	0.017						
	(Age)			0.16	1.34	0.187						
	(Sex)			-0.05	-0.44	0.660						
	(Study Site)			-0.06	-0.41	0.687						

Model 2	**Predictor**	**B**	**SE**	**Beta**	***t***	***p***	**adj-*r2***	***F***	***p***	***r2* change**	***F change***	***F Change p***
Step 1	Constant	10.69	13.95		0.77	0.446	0.13	3.56	0.011	0.08	5.99	0.017
	Diagnostic Group	9.70	14.80	0.16	0.66	0.515						
	PSS	-2.88	0.92	-0.96	-3.13	0.003						
	Mean Glu CR	-4.11	2.14	-0.22	-1.93	0.058						
Step 2	PSS x Diagnostic Group	3.31	1.35	0.75	2.45	0.017						
	(Age)			0.11	0.97	0.335						
	(Sex)			0.02	0.15	0.884						
	(Study Site)			-0.08	-0.63	0.531						

Diagnostic Group x PSS interaction in individuals who completed the acute stress manipulation (Glx)

Model 1	**Predictor**	**B**	**SE**	**Beta**	***t***	***p***	**adj-*r2***	***F***	***p***	***r2* change**	***F change***	***F Change p***
Step 1	Constant	15.85	12.98		1.22	0.226	0.05	2.15	0.102	0.09	2.15	0.102
	Diagnostic Group	22.52	13.59	0.39	1.66	0.102						
	PSS	-1.27	0.66	-0.45	-1.93	0.058						
	Mean Glu CR	-3.50	2.08	-0.20	-1.68	0.098						
Step 2	(PSS x Diagnostic Group)			0.84	2.76	0.007						
	(Age)			0.13	1.05	0.300						
	(Sex)			-0.05	-0.43	0.669						
	(Study Site)			-0.06	-0.42	0.679						

Model 2	**Predictor**	**B**	**SE**	**Beta**	***t***	***p***	**adj-*r2***	***F***	***p***	***r2* change**	***F change***	***F Change p***
Step 1	Constant	5.07	12.97		0.39	0.697	0.13	3.68	0.009	0.10	7.62	0.007
	Diagnostic Group	9.64	13.77	0.17	0.70	0.486						
	PSS	-2.88	0.86	-1.03	-3.36	0.001						
	Mean Glu CR	-3.27	1.99	-0.19	-1.65	0.105						
Step 2	PSS x Diagnostic Group	3.47	1.26	0.84	2.76	0.007						
	(Age)			0.07	0.63	0.532						
	(Sex)			0.03	0.24	0.815						
	(Study Site)			-0.09	-0.67	0.503						

Note. Diagnostic group, PSS, and Cramér-Rao lower bound of glutamate (Mean Glu CR) were entered in the first block and all other predictors were included in the second block with stepwise selection. Model coefficients (adjusted r^2, F) are based on included variables, while t and p are provided for excluded variables. Variables excluded for each model are indicated with parenthesis. Models were run independently (two-tailed) and no corrections for multiple comparisons were applied across models. Glu = Glutamate; Glx = Glutamate + Glutamine; SE = Standard Error; PSS = Perceived Stress Scale.

Supplementary Table 7: Linear vs quadratic effects of PSS on %ΔGlu and %ΔGlx after acute stress.

Quadratic Effect of PSS (Glu)

Model 1	**Predictor**	**B**	**SE**	**Beta**	***t***	***p***	**adj-*r2***	***F***	***p***	***r2* change**	***F change***	***F Change p***
Step 1	Constant	10.46	6.54		1.60	0.114	0.01	1.78	0.186	0.03	1.78	0.186
	PSS	-0.48	0.36	-0.16	-1.34	0.186						
Step 2	(PSS-Squared)			1.17	2.42	0.018						

Model 2	**Predictor**	**B**	**SE**	**Beta**	***t***	***p***	**adj-*r2***	***F***	***p***	***r2* change**	***F change***	***F Change p***
Step 1	Constant	33.02	11.26		2.93	0.005	0.08	3.88	0.025	0.08	5.86	0.018
	PSS	-3.89	1.45	-1.30	-2.68	0.009						
Step 2	PSS-Squared	0.09	0.04	1.17	2.42	0.018						

Quadratic Effect of PSS (Glx)

Model 1	**Predictor**	**B**	**SE**	**Beta**	***t***	***p***	**adj-*r2***	***F***	***p***	***r2* change**	***F change***	***F Change p***
Step 1	Constant	8.61	6.13		1.41	0.165	0.00	1.18	0.281	0.02	1.18	0.281
	PSS	-0.37	0.34	-0.13	-1.09	0.281						
Step 2	(PSS-Squared)			1.26	2.61	0.011						

Model 2	**Predictor**	**B**	**SE**	**Beta**	***t***	***p***	**adj-*r2***	***F***	***p***	***r2* change**	***F change***	***F Change p***
Step 1	Constant	31.25	10.49		2.98	0.004	0.08	4.04	0.022	0.09	6.80	0.011
	PSS	-3.79	1.35	-1.36	-2.80	0.007						
Step 2	PSS-Squared	0.09	0.04	1.26	2.61	0.011						

Note. PSS was entered in the first block and squared PSS term was entered into the second block. Model coefficients (adjusted r^2, F) are based on included variables, while t and p are provided for excluded variables. Variables excluded for each model are indicated with parenthesis. Models were run independently (two-tailed) and no corrections for multiple comparisons were applied across models. Glu = Glutamate; Glx = Glutamate + Glutamine; SE = Standard Error; PSS = Perceived Stress Scale.

Supplementary Table 8: Linear vs quadratic effects of PSS on %ΔGlu and %ΔGlx after acute stress controlling for mean Cramér-Rao lower bound (CRLB) of glutamate.

Quadratic Effect of PSS (Glu)

Model 1	**Predictor**	**B**	**SE**	**Beta**	***t***	***p***	**adj-*r2***	***F***	***p***	***r2* change**	***F change***	***F Change p***
Step 1	Constant	33.47	14.06		2.38	0.020	0.04	2.62	0.080	0.07	2.62	0.080
	PSS	-0.43	0.35	-0.14	-1.21	0.230						
	Mean Glu CR	-4.10	2.23	-0.22	-1.84	0.070						
Step 2	(PSS-Squared)			1.17	2.46	0.016						
Model 2	**Predictor**	**B**	**SE**	**Beta**	***t***	***p***	**adj-*r2***	***F***	***p***	***r2* change**	***F change***	***F Change p***
Step 1	Constant	55.97	16.34		3.43	0.001	0.11	3.90	0.013	0.08	6.07	0.016
	PSS	-3.84	1.42	-1.28	-2.69	0.009						
	Mean Glu CR	-4.10	2.15	-0.22	-1.91	0.061						
Step 2	PSS-Squared	0.09	0.04	1.17	2.46	0.016						

Quadratic Effect of PSS (Glx)

Model 1	**Predictor**	**B**	**SE**	**Beta**	***t***	***p***	**adj-*r2***	***F***	***p***	***r2* change**	***F change***	***F Change p***
Step 1	Constant	26.90	13.27		2.03	0.047	0.02	1.80	0.173	0.05	1.80	0.173
	PSS	-0.33	0.33	-0.12	-0.97	0.334						
	Mean Glu CR	-3.26	2.10	-0.19	-1.55	0.126						
Step 2	(PSS-Squared)			1.26	2.64	0.010						
Model 2	**Predictor**	**B**	**SE**	**Beta**	***t***	***p***	**adj-*r2***	***F***	***p***	***r2* change**	***F change***	***F Change p***
Step 1	Constant	49.48	15.33		3.23	0.002	0.10	3.63	0.017	0.09	6.95	0.010
	PSS	-3.75	1.34	-1.34	-2.80	0.007						
	Mean Glu CR	-3.25	2.02	-0.19	-1.61	0.111						
Step 2	PSS-Squared	0.09	0.03	1.26	2.64	0.010						

Note. PSS and Cramér-Rao lower bound (CRLB) of glutamate (Mean Glu CR) were entered in the first block and squared PSS term was entered into the second block. Model coefficients (adjusted r^2, F) are based on included variables, while t and p are provided for excluded variables. Variables excluded for each model are indicated with parenthesis. Models were run independently (two-tailed) and no corrections for multiple comparisons were applied across models. Glu = Glutamate; Glx = Glutamate + Glutamine; SE = Standard Error; PSS = Perceived Stress Scale.

Supplementary Table 9: Mean Cr ratios and Cramér-Rao lower bounds (CRLB) for MRS metabolites pre-stressor and post-stressor in the healthy control stress sample.

Aspartate (Asp)								
Pre-stressor				Post-Stressor				Paired t-test p-value
Mean ratio (Cr)	SD ratio (Cr)	Mean CRLB	SD CRLB	Mean ratio (Cr)	SD ratio (Cr)	Mean CRLB	SD CRLB	
0.41	0.10	11.36	2.78	0.42	0.10	10.96	2.11	.48
**Choline Containing Metabolites (Primarily Glycerophosphocholine and Phosphocholine; Cho)**								
Pre-stressor				Post-Stressor				Paired t-test p-value
Mean ratio (Cr)	SD ratio (Cr)	Mean CRLB	SD CRLB	Mean ratio (Cr)	SD ratio (Cr)	Mean CRLB	SD CRLB	
.89	.33	4.24	.78	.94	.27	4.08	.64	.48
**Glutamate (Glu)**								
Pre-stressor				Post-Stressor				Paired t-test p-value
Mean ratio (Cr)	SD ratio (Cr)	Mean CRLB	SD CRLB	Mean ratio (Cr)	SD ratio (Cr)	Mean CRLB	SD CRLB	
.96	.16	5.80	1.47	.97	.17	5.92	2.20	.76
**Glutamine (Gln)**								
Pre-stressor				Post-Stressor				Paired t-test p-value
Mean ratio (Cr)	SD ratio (Cr)	Mean CRLB	SD CRLB	Mean ratio (Cr)	SD ratio (Cr)	Mean CRLB	SD CRLB	
.30	.07	13.80	2.02	.30	.11	13.56	3.12	.92
**Myo-inositol (ml)**								
Pre-stressor				Post-Stressor				Paired t-test p-value
Mean ratio (Cr)	SD ratio (Cr)	Mean CRLB	SD CRLB	Mean ratio (Cr)	SD ratio (Cr)	Mean CRLB	SD CRLB	
.63	.21	5.12	1.36	.70	.30	5.48	1.42	.27
**N-Acetylaspartic Acid (NAA) + N-acetylaspartylglutamate (NAAG)**								
Pre-stressor				Post-Stressor				Paired t-test p-value
Mean ratio (Cr)	SD ratio (Cr)	Mean CRLB	SD CRLB	Mean ratio (Cr)	SD ratio (Cr)	Mean CRLB	SD CRLB	
1.10	.13	2.84	.75	1.16	.14	3.00	.96	.04
**Glx (Glutamate + Glutamine)**								
Pre-stressor				Post-Stressor				Paired t-test p-value
Mean ratio (Cr)	SD ratio (Cr)	Mean CRLB	SD CRLB	Mean ratio (Cr)	SD ratio (Cr)	Mean CRLB	SD CRLB	
1.26	.19	-	-	1.27	.20	-	-	.84

Note: All paired *t*-tests are two-tailed, uncorrected. Cr = Creatine-containing metabolites (creatine and phosphocreatine); MRS = magnetic resonance spectroscopy. Source data are provided as a Source Data file.

Supplementary Table 10: Mean Cr ratios and Cramér-Rao lower bounds (CRLB) for MRS metabolites pre-stressor and post-stressor in the healthy control stress replication sample.

Aspartate (Asp)								
Pre-stressor				Post-stressor				Paired t-test p-value
Mean ratio (Cr)	SD ratio (Cr)	Mean CRLB	SD CRLB	Mean ratio (Cr)	SD ratio (Cr)	Mean CRLB	SD CRLB	
.49	.10	9.09	2.49	.48	.09	9.50	1.95	.40

Choline Containing Metabolites (Primarily Glycerophosphocholine and Phosphocholine; Cho)								
Pre-stressor				Post-stressor				Paired t-test p-value
Mean ratio (Cr)	SD ratio (Cr)	Mean CRLB	SD CRLB	Mean ratio (Cr)	SD ratio (Cr)	Mean CRLB	SD CRLB	
.72	.11	3.86	.64	.75	.13	4.27	.70	.32

Glutamate (Glu)								
Pre-stressor				Post-stressor				Paired t-test p-value
Mean ratio (Cr)	SD ratio (Cr)	Mean CRLB	SD CRLB	Mean ratio (Cr)	SD ratio (Cr)	Mean CRLB	SD CRLB	
.96	.21	5.64	2.06	.97	.26	5.36	2.08	.88

Glutamine (Gln)								
Pre-stressor				Post-stressor				Paired t-test p-value
Mean ratio (Cr)	SD ratio (Cr)	Mean CRLB	SD CRLB	Mean ratio (Cr)	SD ratio (Cr)	Mean CRLB	SD CRLB	
.22	.04	12.14	2.59	.21	.05	12.86	3.26	.72

Myo-inositol (ml)								
Pre-stressor				Post-stressor				Paired t-test p-value
Mean ratio (Cr)	SD ratio (Cr)	Mean CRLB	SD CRLB	Mean ratio (Cr)	SD ratio (Cr)	Mean CRLB	SD CRLB	
.80	.14	4.45	1.26	.80	.09	4.00	.98	.85

N-Acetylaspartic Acid (NAA) + N-acetylaspartylglutamate (NAAG)								
Pre-stressor				Post-stressor				Paired t-test p-value
Mean ratio (Cr)	SD ratio (Cr)	Mean CRLB	SD CRLB	Mean ratio (Cr)	SD ratio (Cr)	Mean CRLB	SD CRLB	
1.06	.23	3.05	1.40	.98	.32	3.41	1.74	.05

Glx (Glutamate + Glutamine)								
Pre-stressor				Post-stressor				Paired t-test p-value
Mean ratio (Cr)	SD ratio (Cr)	Mean CRLB	SD CRLB	Mean ratio (Cr)	SD ratio (Cr)	Mean CRLB	SD CRLB	
1.18	.22	-	-	1.18	.28	-	-	.95

Note: This sample contains two influential datapoint for percent change NAA (>2.5 SD). While the paired *t*-test for NAA is significant in the whole sample (p = .05), it is not significant when these two datapoints are excluded, t_{19} = 1.409, p = .175. All paired *t*-tests are two-tailed, uncorrected. Cr = Creatine-containing metabolites (creatine and phosphocreatine); MRS = magnetic resonance spectroscopy. Source data are provided as a Source Data file.

Supplementary Table 11: Mean Cr ratios and Cramér-Rao lower bounds (CRLB) for MRS metabolites pre-no stress control (NSC) and post-NSC in the no stress control sample.

Aspartate (Asp)								
Pre-NSC				Post-NSC				Paired t-test p-value
Mean ratio (Cr)	SD ratio (Cr)	Mean CRLB	SD CRLB	Mean ratio (Cr)	SD ratio (Cr)	Mean CRLB	SD CRLB	
.42	.09	11.83	3.43	.42	.09	11.06	4.14	.78
**Choline Containing Metabolites (Primarily Glycerophosphocholine and Phosphocholine; Cho)**								
Pre-NSC				Post-NSC				Paired t-test p-value
Mean ratio (Cr)	SD ratio (Cr)	Mean CRLB	SD CRLB	Mean ratio (Cr)	SD ratio (Cr)	Mean CRLB	SD CRLB	
.71	.07	4.56	1.42	.72	.11	4.61	1.38	.92
**Glutamate (Glu)**								
Pre-NSC				Post-NSC				Paired t-test p-value
Mean ratio (Cr)	SD ratio (Cr)	Mean CRLB	SD CRLB	Mean ratio (Cr)	SD ratio (Cr)	Mean CRLB	SD CRLB	
.98	.21	5.83	2.07	.94	.16	6.56	1.62	.30
**Glutamine (Gln)**								
Pre-NSC				Post-NSC				Paired t-test p-value
Mean ratio (Cr)	SD ratio (Cr)	Mean CRLB	SD CRLB	Mean ratio (Cr)	SD ratio (Cr)	Mean CRLB	SD CRLB	
.25	.08	13.89	2.89	.24	.09	13.67	3.51	.52
**Myo-inositol (ml)**								
Pre-NSC				Post-NSC				Paired t-test p-value
Mean ratio (Cr)	SD ratio (Cr)	Mean CRLB	SD CRLB	Mean ratio (Cr)	SD ratio (Cr)	Mean CRLB	SD CRLB	
.84	.15	4.61	1.14	.81	.16	4.50	.99	.45
**N-Acetylaspartic Acid (NAA) + N-acetylaspartylglutamate (NAAG)**								
Pre-NSC				Post-NSC				Paired t-test p-value
Mean ratio (Cr)	SD ratio (Cr)	Mean CRLB	SD CRLB	Mean ratio (Cr)	SD ratio (Cr)	Mean CRLB	SD CRLB	
1.03	.17	3.33	1.57	1.00	.18	3.61	1.29	.24
**Glx (Glutamate + Glutamine)**								
Pre-NSC				Post-NSC				Paired t-test p-value
Mean ratio (Cr)	SD ratio (Cr)	Mean CRLB	SD CRLB	Mean ratio (Cr)	SD ratio (Cr)	Mean CRLB	SD CRLB	
1.24	.26	-	-	1.18	.23	-	-	.32

Note: All paired *t*-tests are two-tailed, uncorrected. Cr = Creatine-containing metabolites (creatine and phosphocreatine); MRS = magnetic resonance spectroscopy. Source data are provided as a Source Data file.

Supplementary Table 12: Mean Cr ratios and Cramér-Rao lower bounds (CRLB) for MRS metabolites pre-stressor and post-stressor in the sample of participants with major depressive disorder.

Aspartate (Asp)								
Pre-stressor				Post-Stressor				Paired t-test p-value
Mean ratio (Cr)	SD ratio (Cr)	Mean CRLB	SD CRLB	Mean ratio (Cr)	SD ratio (Cr)	Mean CRLB	SD CRLB	
.47	.11	10.87	2.32	.48	.11	10.70	2.53	.47
**Choline Containing Metabolites (Primarily Glycerophosphocholine and Phosphocholine; Cho)**								
Pre-stressor				Post-Stressor				Paired t-test p-value
Mean ratio (Cr)	SD ratio (Cr)	Mean CRLB	SD CRLB	Mean ratio (Cr)	SD ratio (Cr)	Mean CRLB	SD CRLB	
.71	.10	4.22	.80	.80	.22	4.22	.95	.06
**Glutamate (Glu)**								
Pre-stressor				Post-Stressor				Paired t-test p-value
Mean ratio (Cr)	SD ratio (Cr)	Mean CRLB	SD CRLB	Mean ratio (Cr)	SD ratio (Cr)	Mean CRLB	SD CRLB	
.99	.16	5.61	1.88	.98	.26	6.43	2.13	.81
**Glutamine (Gln)**								
Pre-stressor				Post-Stressor				Paired t-test p-value
Mean ratio (Cr)	SD ratio (Cr)	Mean CRLB	SD CRLB	Mean ratio (Cr)	SD ratio (Cr)	Mean CRLB	SD CRLB	
.24	.06	14.00	2.43	.25	.08	13.61	3.30	.46
**Myo-inositol (ml)**								
Pre-stressor				Post-Stressor				Paired t-test p-value
Mean ratio (Cr)	SD ratio (Cr)	Mean CRLB	SD CRLB	Mean ratio (Cr)	SD ratio (Cr)	Mean CRLB	SD CRLB	
.79	.11	4.61	1.12	.77	.18	4.57	1.04	.71
**N-Acetylaspartic Acid (NAA) + N-acetylaspartylglutamate (NAAG)**								
Pre-stressor				Post-Stressor				Paired t-test p-value
Mean ratio (Cr)	SD ratio (Cr)	Mean CRLB	SD CRLB	Mean ratio (Cr)	SD ratio (Cr)	Mean CRLB	SD CRLB	
1.02	.16	3.00	1.09	.95	.19	3.87	1.14	.05
**Glx (Glutamate + Glutamine)**								
Pre-stressor				Post-Stressor				Paired t-test p-value
Mean ratio (Cr)	SD ratio (Cr)	Mean CRLB	SD CRLB	Mean ratio (Cr)	SD ratio (Cr)	Mean CRLB	SD CRLB	
1.23	.20	-	-	1.23	.28	-	-	.98

Note: All paired *t*-tests are two-tailed, uncorrected. Cr = Creatine-containing metabolites (creatine and phosphocreatine); MRS = magnetic resonance spectroscopy. Source data are provided as a Source Data file.

Supplementary Table 13: Comorbidities in participants with major depressive disorder.

Anxiety disorders	
Generalized anxiety disorder (current)	8
Panic disorder (current)	1
Social phobia (current)	5
Social phobia (past)	1
Agoraphobia	1
Substance use and dependence	
Past alcohol abuse	4
Past alcohol dependence	2
Past THC dependence	1
Post-traumatic stress disorder (PTSD)	
PTSD (current)	1
PTSD (past)	3

Note. The number of study completers meeting diagnostic criteria for each comorbidity is shown. Participants are included in the count for every disorder for which they met criteria. All participants were free from psychotropic medications. THC = tetrahydrocannabinol.

Supplementary Table 14: Hierarchical regression models testing stress by age and stress by sex interactions on %ΔGlu and %ΔGlx in healthy controls.

Sex interaction with stress in healthy control stress samples (Glu)

Model 1	**Predictor**	**B**	**SE**	**Beta**	***t***	***p***	**adj-*r2***	***F***	***p***	***r2* change**	***F change***	***F Change p***
Step 1	Constant	4.80	4.85		0.99	0.328	0.151	5.10	0.010	0.19	5.10	0.010
	PSS	-2.85	0.96	-0.42	-2.99	0.005						
	Sex	-1.78	8.85	-0.03	-0.20	0.842						
Step 2	(PSS by Sex)			0.16	0.93	0.355						
	(Study Site)			-0.07	-0.48	0.632						

Age interaction with stress in healthy control stress samples (Glu)

Model 1	**Predictor**	**B**	**SE**	**Beta**	***t***	***p***	**adj-*r2***	***F***	***p***	***r2* change**	***F change***	***F Change p***
Step 1	Constant	4.23	3.77		1.12	0.269	0.223	7.59	0.001	0.26	7.59	0.001
	PSS	-2.42	0.91	-0.36	-2.67	0.011						
	Age	1.11	0.55	0.27	2.02	0.049						
Step 2	(PSS by Age)			0.12	0.84	0.408						
	(Study Site)			-0.10	-0.74	0.462						

Sex interaction with stress in healthy control stress samples (Glx)

Model 1	**Predictor**	**B**	**SE**	**Beta**	***t***	***p***	**adj-*r2***	***F***	***p***	***r2* change**	***F change***	***F Change p***
Step 1	Constant	4.10	4.43		0.92	0.361	0.181	6.08	0.005	0.22	6.08	0.005
	PSS	-2.83	0.87	-0.45	-3.24	0.002						
	Sex	-2.20	8.09	-0.04	-0.27	0.787						
Step 2	(PSS by Sex)			0.27	1.62	0.112						
	(Study Site)			-0.08	-0.57	0.569						

Age interaction with stress in healthy control stress samples (Glx)

Model 1	**Predictor**	**B**	**SE**	**Beta**	***t***	***p***	**adj-*r2***	***F***	***p***	***r2* change**	***F change***	***F Change p***
Step 1	Constant	3.39	3.49		0.97	0.336	0.231	7.92	0.001	0.27	7.92	0.001
	PSS	-2.51	0.84	-0.40	-3.00	0.004						
	Age	0.87	0.51	0.23	1.72	0.092						
Step 2	(PSS by Age)			0.14	0.97	0.335						
	(Study Site)			-0.10	-0.78	0.440						

Note. For each model, PSS and the main demographic variable of interest were entered in the first block and interaction terms were included in the second block with stepwise selection. Model coefficients (adjusted r^2, F) are based on included variables, while t and p are provided for excluded variables. Variables excluded for each model are indicated with parenthesis. Models were run independently (two-tailed) and no corrections for multiple comparisons were applied across models. Glu = Glutamate; Glx = Glutamate + Glutamine; SE = Standard Error; PSS = Perceived Stress Scale.

Supplementary Table 15: Hierarchical regression models testing effects of birth control on %∆Glu and %∆Glx in healthy control female participants.

Birth control interaction with PSS in female healthy controls following acute stress (Glu)

Model 1	**Predictor**	**B**	**SE**	**Beta**	***t***	***p***	**adj-*r2***	***F***	***p***	***r2* change**	***F change***	***F Change p***
Step 1	Constant	6.15	6.63		0.93	0.361	0.16	3.88	0.032	0.21	3.88	0.032
	PSS	-3.57	1.29	-0.46	-2.78	0.009						
	Birth Control (No/Yes)	2.82	10.47	0.05	0.27	0.789						
Step 2	(Birth Control x PSS)			0.01	0.06	0.952						
	(Study Site)			-0.09	-0.49	0.629						

Birth control interaction with PSS in female healthy controls following acute stress (Glx)

Model 1	**Predictor**	**B**	**SE**	**Beta**	***t***	***p***	**adj-*r2***	***F***	***p***	***r2* change**	***F change***	***F Change p***
Step 1	Constant	7.86	5.60		1.40	0.171	0.26	6.57	0.004	0.31	6.57	0.004
	PSS	-3.81	1.09	-0.55	-3.51	0.001						
	Birth Control (No/Yes)	-3.16	8.85	-0.06	-0.36	0.724						
Step 2	(Birth Control x PSS)			0.07	0.39	0.701						
	(Study Site)			-0.10	-0.65	0.524						

Note. PSS and birth control (no/yes) were entered in the first block and birth control x PSS interaction term was included in the second block with stepwise selection. Model coefficients (adjusted r^2, *F*) are based on included variables, while *t* and *p* are provided for excluded variables. Variables excluded for each model are indicated with parenthesis. Models were run independently (two-tailed) and no corrections for multiple comparisons were applied across models. Glu = Glutamate; Glx = Glutamate + Glutamine; SE = Standard Error; PSS = Perceived Stress Scale.

Supplementary Figure 1: Changes in creatine-normalized mPFC Glx (Glutamate + Glutamine) in response to acute and perceived stress.

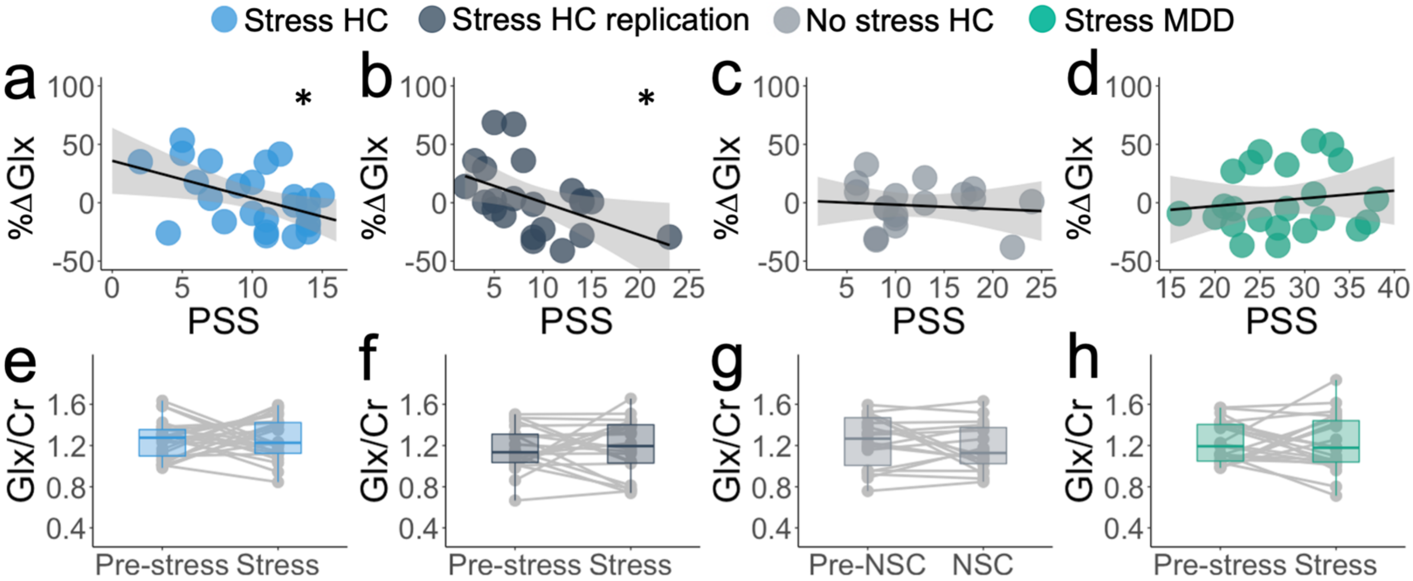

(**a**) Association between perceived stress (PSS scores) and percent change in MRS Glx/Cr signal ($r_{s(23)}$ = -.401, p = .047, two-tailed, uncorrected) in healthy control stress sample. (**b**) Association between perceived stress (PSS scores) and percent change in MRS Glx/Cr signal ($r_{s(20)}$ = -.465, p = .029, two-tailed, uncorrected) in the healthy control stress replication sample. (**c**) Association between perceived stress (PSS scores) and percent change in MRS Glx/Cr signal ($r_{s(16)}$ = -.087, p = .730, two-tailed, uncorrected) in no stress control sample. (**d**) Association between perceived stress (PSS scores) and percent change in MRS Glx/Cr signal ($r_{s(21)}$ = .115, p = .600, two-tailed, uncorrected) in participants with major depressive disorder. Shaded area on **a-d** represents 95% confidence interval, *p<.05. **e-h.** Glx/Cr ratios before and after MAST/NSC in (**e**) healthy control stress sample (n = 25 participants), (**f**) healthy control stress replication (n = 22 participants), (**g**) no stress control (n = 18 participants), and (**h**) participants with major depressive disorder (n = 23 participants). Box plot elements indicate median (center line), first and third quartiles (box limits; 25–75th percentile), smallest observation within 1.5 times the interquartile range from the lower quartile (bottom whisker), largest observation within 1.5 times the interquartile range from the upper quartile (top whisker), and all individual participants (points). Cr = Creatine-containing metabolites (Creatine and Phosphocreatine); Glx = glutamine/glutamate/glutathione; HC = healthy control; MAST = Maastricht Acute Stress Test; MDD = participants with major depressive disorder; NSC = no stress control; PSS = Perceived Stress Scale. Source data are provided as a Source Data file.

Supplementary Figure 2: Effects of acute stress on glutamate and NAA in healthy control participants with low perceived stress.

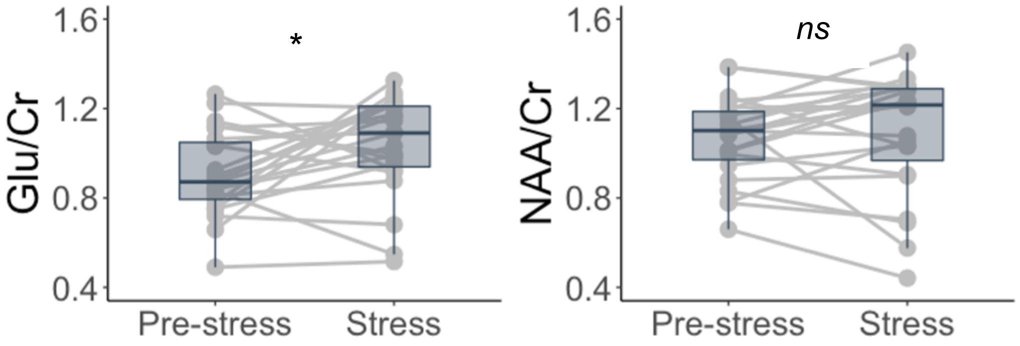

Effects of acute stress on creatine-normalized glutamate (left) and NAA (right) for participants with perceived stress scale (PSS) scores < 10 (*n* = 23 participants). Glu/Cr increased with stress, paired-t_{22} = 2.39, *p* = .026 (two-tailed, uncorrected), while NAA/Cr did not change, paired-t_{22} = .525, *p* = .605 (two-tailed, uncorrected). NAA = N-acetylaspartic acid and N-acetylaspartylglutamate; Glu = glutamate; Cr = creatine and phosphocreatine. Box plot elements indicate median (center line), first and third quartiles (box limits; 25–75th percentile), smallest observation within 1.5 times the interquartile range from the lower quartile (bottom whisker), largest observation within 1.5 times the interquartile range from the upper quartile (top whisker), and all individual participants (points). *p<.05; *ns* = not significant (p > .05). Source data are provided as a Source Data file.

<u>Supplementary Figure 3</u>: Intraclass correlation coefficients.

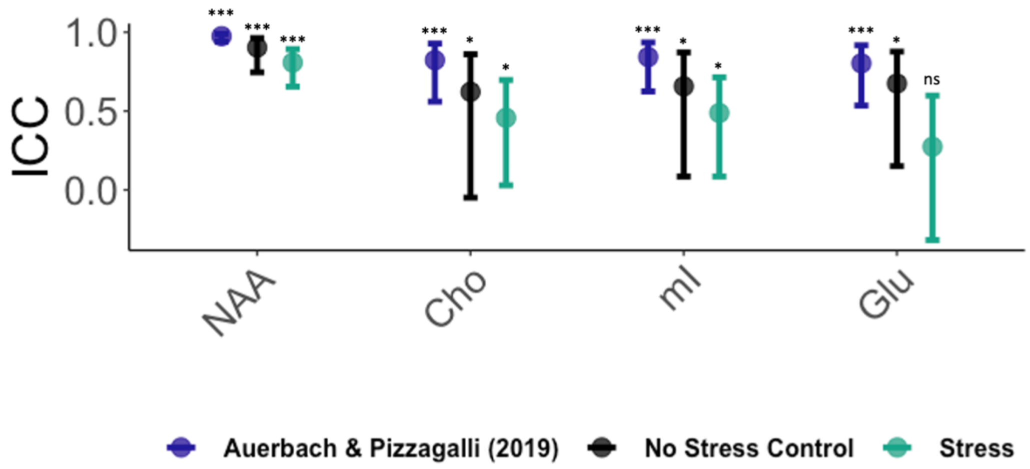

Intraclass correlation coefficients (ICCs) are included for a published test-rest sample (Auerbach & Pizzagalli, 2019, *n* = 22 participants), no stress control sample (*n* = 18 participants), and healthy control stress samples ("stress"; *n* = 47 participants) and were calculated using two-way mixed models with absolute agreement (uncorrected). Error bars represent 95% confidence intervals. The ICC values and *p* values for Auerbach & Pizzagalli (2019), no stress control, and stress conditions, respectively, were: ICC = .975, $p < .001$; ICC = .903, $p < .001$; ICC = .808, $p < .001$ for NAA, ICC = .823, $p < .001$; ICC = .622, $p = .031$; ICC = .457, $p = .020$ for Cho, ICC = .843, $p < .001$; ICC = .657, $p = .018$; ICC = .488, $p = .012$ for mI, and ICC = .803, $p < .001$; ICC = .675, $p = .013$; and ICC = .274, $p = .145$ for Glu. NAA = N-acetylaspartic acid and N-acetylaspartylglutamate; Cho = Choline-containing metabolites (primarily glycerophosphocholine + phosphocholine); mI = myo-inositol; Glu = glutamate. ***$p<.001$, **$p<.01$, *$p<.05$, ns = not significant ($p > .05$). Source data for Stress and No Stress Control groups are provided as a Source Data file. Data shown for the test-retest sample were reproduced from results published in Table 1 of Auerbach & Pizzagalli (2019).

<u>Supplementary Figure 4:</u> Association between age and baseline Glu/Cr in all participants.

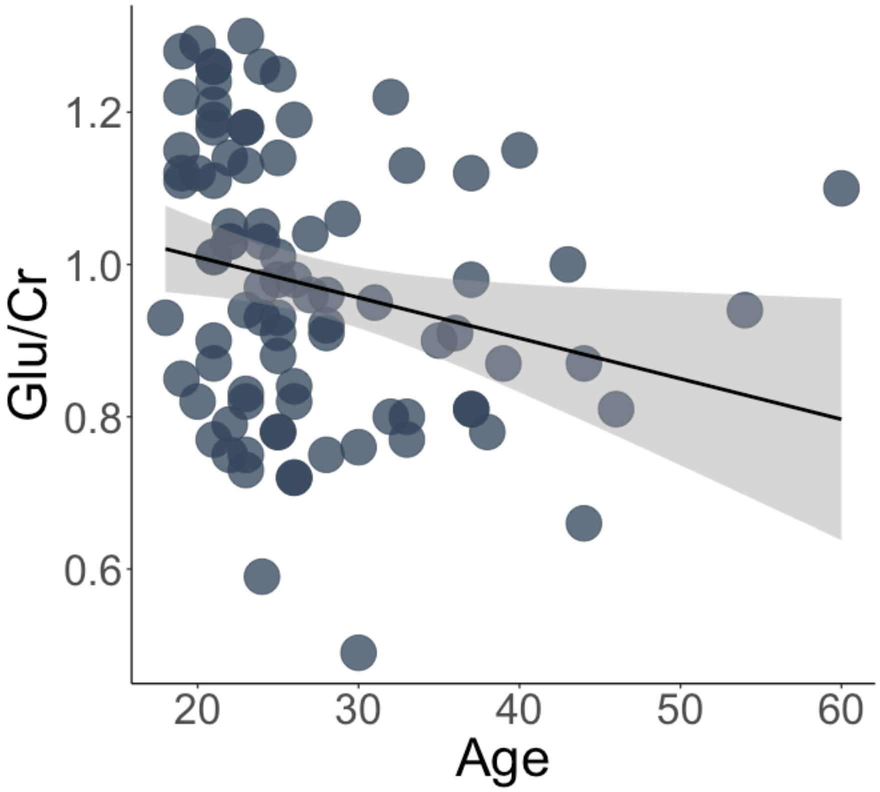

Glu/Cr is negatively correlated with age, r_{86} = -.237, p = .026. Shaded area represents 95% confidence interval. Glu = glutamate; Cr = creatine and phosphocreatine. Source data are provided as a Source Data file.

<u>Supplementary Figure 5:</u> Relative frequencies of accurate and inaccurate expectations from ecological momentary assessment (EMA) data.

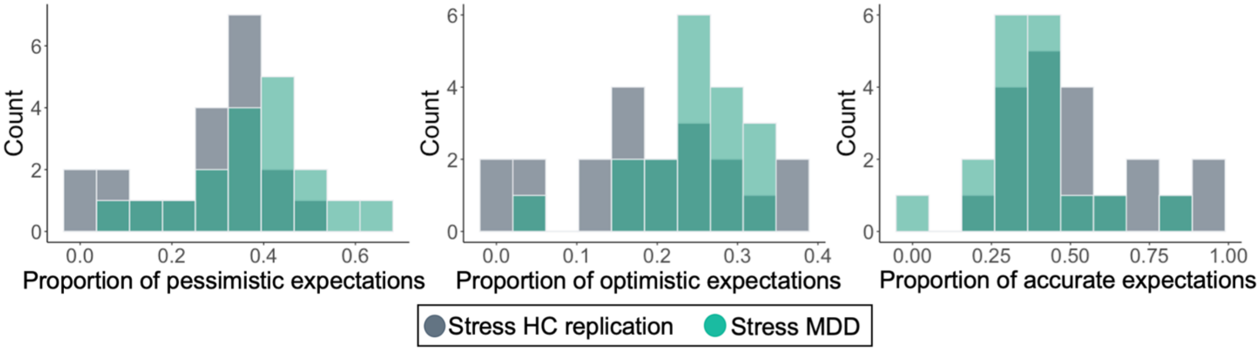

The proportion of observations of each type, relative to total observations, were computed for each participant. Histograms represent the distribution of proportions for pessimistic expectations (left), optimistic expectations (middle), and accurate expectations (right) for the healthy control replication sample (grey; *n* = 20 participants) and participants with major depressive disorder (teal; *n* = 18 participants). HC = healthy control participants; MDD = participants with major depressive disorder. Source data are provided as a Source Data file.

Supplementary Figure 6: Test-retest of MRS acquisition sequence at Emory University.

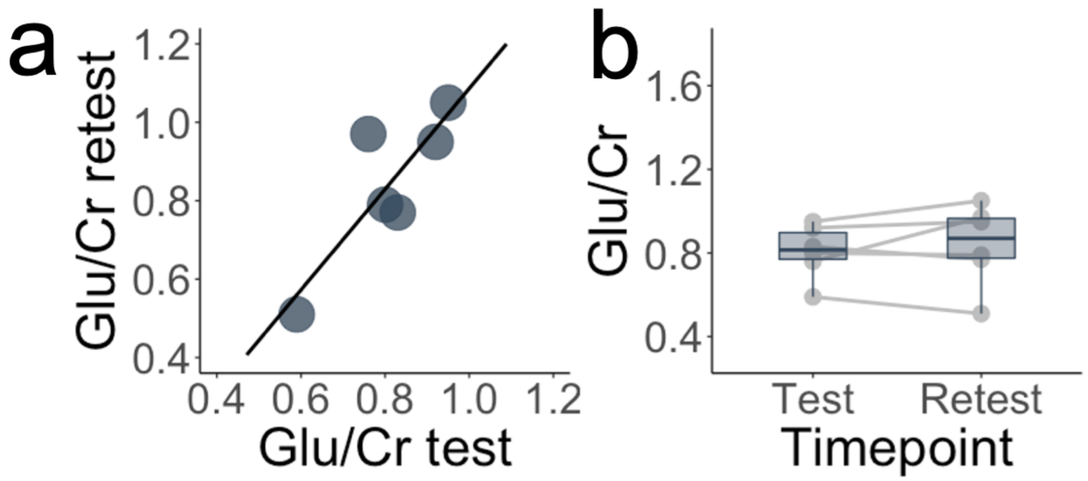

(**a**) Scatter plot for test-retest MRS data collected in six participants during the same scanning session with no stress manipulation in between MRS acquisitions (Intra-class correlation coefficient (ICC) = 0.89, p = .017; two-way mixed with absolute agreement, uncorrected). These data were collected at Emory University using the same Siemens Tim Trio and identical MRS protocol. (**b**) Box plot showing reliability of test-retest data. Glu/Cr at test (M = .81, SEM = .05) was not significantly different from Glu/Cr at retest (M = .84, SEM = .08; t_5 = .706, p = .52 (two-tailed, uncorrected); n = 6 participants). Box plot elements indicate median (center line), first and third quartiles (box limits; 25–75th percentile), smallest observation within 1.5 times the interquartile range from the lower quartile (bottom whisker), largest observation within 1.5 times the interquartile range from the upper quartile (top whisker), and all individual participants (points). Cr = Creatine-containing metabolites (Creatine and Phosphocreatine); Glu = glutamate; SEM = standard error of the mean. Source data are provided as a Source Data file.

__Supplementary Figure 7:__ **Ecological momentary assessment (EMA) items and survey flow.**

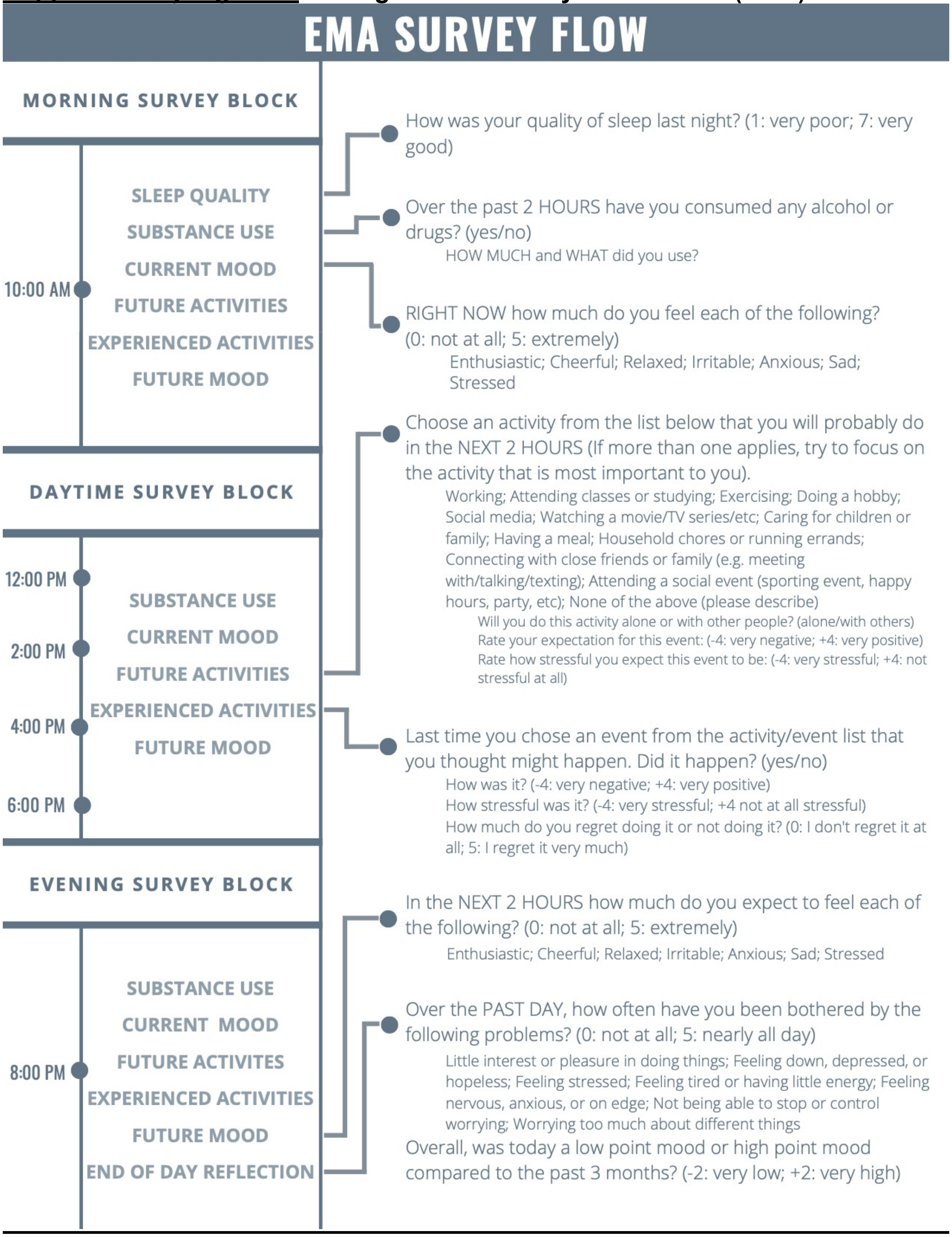

Individual questions (left) were sent 6 times per day, every other day, according to survey flow (right). Median start time was 10am and ranged from 6am to 12pm, depending on the participant's preferred schedule.

Supplementary References

1. Dickerson, S.S. & Kemeny, M.E. Acute stressors and cortisol responses: a theoretical integration and synthesis of laboratory research. *Psychol. Bull.* **130**, 355-391 (2004).
2. Houtepen, L.C. et al. Acute stress effects on GABA and glutamate levels in the prefrontal cortex: A 7T (1)H magnetic resonance spectroscopy study. *Neuroimage Clin* **14**, 195-200 (2017).
3. Auerbach, R.P. & Pizzagalli, D.A. Localized MRS reliability of in vivo glutamate at 3 T in shortened scan times: A feasibility study-Efforts to improve rigor and reproducibility. *NMR Biomed.* **32**, e4093 (2019).
4. Marsman, A. et al. Glutamate changes in healthy young adulthood. *Eur. Neuropsychopharmacol.* **23**, 1484-1490 (2013).
