# Supplementary material for: Reduced adaptation of glutamatergic stress response is associated with pessimistic expectations in depression
Source: Nat Commun. 2021 May 26;12:3166. doi: 10.1038/s41467-021-23284-9 (PMC8155144; doi:10.1038/s41467-021-23284-9)
Supplement: Supplementary file 2 — Reporting Summary [file 41467_2021_23284_MOESM2_ESM.pdf]

nature research

Corresponding author(s): Michael T. Treadway
Last updated by author(s): 1/20/2021

Reporting Summary

Nature Research wishes to improve the reproducibility of the work that we publish. This form provides structure for consistency and transparency in reporting. For further information on Nature Research policies, see our Editorial Policies and the Editorial Policy Checklist.

Statistics

For all statistical analyses, confirm that the following items are present in the figure legend, table legend, main text, or Methods section.

n/a	Confirmed	
☐	☒	The exact sample size (*n*) for each experimental group/condition, given as a discrete number and unit of measurement
☒	☐	A statement on whether measurements were taken from distinct samples or whether the same sample was measured repeatedly
☐	☒	The statistical test(s) used AND whether they are one- or two-sided<br>*Only common tests should be described solely by name; describe more complex techniques in the Methods section.*
☐	☒	A description of all covariates tested
☐	☒	A description of any assumptions or corrections, such as tests of normality and adjustment for multiple comparisons
☐	☒	A full description of the statistical parameters including central tendency (e.g. means) or other basic estimates (e.g. regression coefficient) AND variation (e.g. standard deviation) or associated estimates of uncertainty (e.g. confidence intervals)
☐	☒	For null hypothesis testing, the test statistic (e.g. *F*, *t*, *r*) with confidence intervals, effect sizes, degrees of freedom and *P* value noted<br>*Give P values as exact values whenever suitable.*
☒	☐	For Bayesian analysis, information on the choice of priors and Markov chain Monte Carlo settings
☐	☒	For hierarchical and complex designs, identification of the appropriate level for tests and full reporting of outcomes
☐	☒	Estimates of effect sizes (e.g. Cohen's *d*, Pearson's *r*), indicating how they were calculated

Our web collection on statistics for biologists contains articles on many of the points above.

Software and code

Policy information about availability of computer code

Data collection	MRS data collection used a proton MRS sequence developed by J. Eric Jensen.
Data analysis	Data analysis used commercially available and open source programs, including Matlab 2013B (Mathworks, Natick, MA), SPSS v27 (IBM, Armonk, NY), R v3.6.0, and Jupyter Notebooks 4.4.0 in Python 3.7.1. MRS data were processed and analyzed using jMRUI 5.2 and LCModel version 6.3-1K. Ecological momentary assessment data was collected using Qualtrics XM Survey Software.

For manuscripts utilizing custom algorithms or software that are central to the research but not yet described in published literature, software must be made available to editors and reviewers. We strongly encourage code deposition in a community repository (e.g. GitHub). See the Nature Research guidelines for submitting code & software for further information.

Data

Policy information about availability of data

All manuscripts must include a data availability statement. This statement should provide the following information, where applicable:

- Accession codes, unique identifiers, or web links for publicly available datasets
- A list of figures that have associated raw data
- A description of any restrictions on data availability

Source data are provided with this paper.

Field-specific reporting

Please select the one below that is the best fit for your research. If you are not sure, read the appropriate sections before making your selection.

☐ Life sciences ☒ Behavioural & social sciences ☐ Ecological, evolutionary & environmental sciences

For a reference copy of the document with all sections, see nature.com/documents/nr-reporting-summary-flat.pdf

Behavioural & social sciences study design

All studies must disclose on these points even when the disclosure is negative.

Study description	This study collected quantitative measures of glutamate using single voxel MR spectroscopy. A repeated-measures design was used to quantify within-subject glutamate response to an acute stressor. A between-subjects design was used to compare stress effects to a no stress control condition and to compare healthy control participants to those with major depressive disorder. Other quantitative variables included self-report surveys and interview measures, salivary cortisol concentration, and data from ecological momentary assessment.
Research sample	To study the effect of stress in healthy adults and adults with major depressive disorder, we recruited participants from the Boston area at McLean Hospital and the Atlanta community through Emory University. Participants (70% female) ranged from 18 to 60 years of age (mean = 27.09), and were 62.5% white/caucasian, 19.3% Asian, and 17.0% black/African American. At both McLean and Emory sites, convenience samples were used.
Sampling strategy	Samples were recruited from a database of participants who filled out an online pre-screening questionnaire and stratified by psychiatric diagnosis into one group with depression and three healthy control groups. Samples were recruited to have a range of perceived stress, as measured by the Perceived Stress Scale, and to have variation in sex , race, and age. Target sample sizes were based on ability to detect an effect size (r) of 0.5.
Data collection	Data collection procedures included neuroimaging data collected on a 3T scanner (MR spectroscopy), self-report and surveys collected on paper (paper and pen), verbally, or on a computer, clinical interviews conducted by a masters level clinician, biological samples (salivary cortisol), and longitudinal data collecting via cell phone. Researchers were not blind to experimental conditions.
Timing	McLean samples were collected between 05/2014 and 01/2016. Emory samples were collected between 12/2016 and 07/2019.
Data exclusions	Data were excluded for analysis based on quality of MRS data, assessed by MR physicist blind to study results. Exclusion criteria for MRS data included signal to noise ratio (SNR) less than 9, full width at half maximum (FWHM) greater than 0.15, Cramer-Rao lower bound for glutamate greater than 20%, or poor spectral quality based on visual inspection. Twenty two subjects had data judged to be of insufficient quality. One additional participant was excluded for a change in glutamate over 3 standard deviations from the mean. Ecological momentary assessment data was excluded from two participants for low response rate.
Non-participation	Thirteen participants did not finish the scan visit due to time constraints, undiagnosed claustrophobia, subject illness, inability to fit comfortably in the scanner, or scanner malfunction.
Randomization	Only participants with depression were included in the depression group, and only healthy control participants were included in the other groups. Some participants were allocated to groups based on timing (e.g. the stress sample of healthy control participants at McLean was recruited before the Emory samples, warm water controls at Emory were recruited first in order to confirm the specificity of the acute stress manipulation, while the replication stress sample and MDD sample were recruited concurrently and assigned to groups based on diagnosis). This strategy was implemented to optimize matching between the stress healthy control and depression groups.

Reporting for specific materials, systems and methods

We require information from authors about some types of materials, experimental systems and methods used in many studies. Here, indicate whether each material, system or method listed is relevant to your study. If you are not sure if a list item applies to your research, read the appropriate section before selecting a response.

Materials & experimental systems

n/a	Involved in the study	
☒	☐	Antibodies
☒	☐	Eukaryotic cell lines
☒	☐	Palaeontology and archaeology
☒	☐	Animals and other organisms
☐	☒	Human research participants
☒	☐	Clinical data
☒	☐	Dual use research of concern

Methods

n/a	Involved in the study	
☒	☐	ChIP-seq
☒	☐	Flow cytometry
☐	☒	MRI-based neuroimaging

Human research participants

Policy information about studies involving human research participants

Population characteristics	Participants (70% female) ranged from 18 to 60 years of age (mean = 27.09), and were 62.5% white/caucasian, 19.3% Asian, and 17.0% black/African American. Participants included three samples of healthy controls with no current or past psychiatric disorder (with the exception of specific phobia, or past alcohol abuse) and one sample of participants with major depressive disorder.
Recruitment	Participants were recruited using community advertisements. Participants were included if they met all described inclusion criteria. We do not expect any potential biases to affect the results.
Ethics oversight	All recruitment and testing procedures were approved by the Partners Institutional Review Board (McLean Hospital) and the Emory University Institutional Review Board

Note that full information on the approval of the study protocol must also be provided in the manuscript.

Magnetic resonance imaging

Experimental design

Design type	Single voxel MR Spectroscopy
Design specifications	NA; no trials/tasks used during MRS aquisition
Behavioral performance measures	NA; no behavioral measures were recorded during MRS acquisition

Acquisition

Imaging type(s)	J-resolved PRESS protocol (2D-JPRESS)
Field strength	3T
Sequence & imaging parameters	Following the additional automated optimization of water suppression power, carrier-frequency, tip angles and coil tuning, the 2D-JPRESS sequence collected 22 echo time (TE)-stepped spectra with the echo-time ranging from 30ms to 350ms in 15ms increments. Acquisition parameters were: repetition time (TR)=2 s, f1 acquisition bandwidth=67 Hz, spectral bandwidth=2 kHz, readout duration=512 ms, NEX=16/TE-step, total scan duration=12 min.
Area of acquisition	Medial prefrontal cortex. A single 2cm x 2cm x 2cm voxel in the mPFC such that the posterior edge of the voxel was placed directly in front of the anterior edge of the corpus callosum.

Diffusion MRI ☐ Used ☒ Not used

Preprocessing

Preprocessing software	Raw DICOM files were imported in jMRUI for visual inspection and conversion to ASCII format for analysis in LCModel. No other preprocessing is required for SVS MRS.
Normalization	NA; this processing step is not applicable for SVS MRS.
Normalization template	NA; this processing step is not applicable for SVS MRS.
Noise and artifact removal	NA; this processing step is not applicable for SVS MRS.
Volume censoring	NA; this processing step is not applicable for SVS MRS.

Statistical modeling & inference

Model type and settings	The 22 TE-stepped free-induction decay (FIDs) were first zero-filled out to 64 points (TE-stepped dimension), Gaussian-filtered, and Fourier transformed. Every J-resolved spectral extraction within a bandwidth of 67 Hz was fitted with LCModel and its theoretically-correct template, which used an optimized GAMMA-simulated J-resolved basis sets modeled for 2.89 T. The integrated area under the entire 2D surface for each metabolite was calculated by summing the raw peak areas across all 64 J-resolved extractions for each metabolite.
Effect(s) tested	Metabolites were expressed as ratios to total creatine (Cr), and were used to calculate percent change. Analyses were conducted for glutamate, glx (glutamate and glutamine) and choline. Associations with percent change (i.e. percent change glutamate) and self-report, demographic, and ecological momentary assessment variables were assessed with bivariate and partial correlations and hierarchical regression. Effects of cortisol and self reports were assessed using ANOVA.

Specify type of analysis: ☐ Whole brain ☒ ROI-based ☐ Both

Anatomical location(s)	A single 2cm x 2cm x 2cm voxel was placed in the mPFC during MRS acquisition such that the posterior edge of the voxel was placed directly in front of the anterior edge of the corpus callosum
Statistic type for inference (See Eklund et al. 2016)	NA; all analyses were based on a single voxel.
Correction	NA; all analyses were based on a single voxel.

Models & analysis

n/a	Involved in the study
☒	☐ Functional and/or effective connectivity
☒	☐ Graph analysis
☒	☐ Multivariate modeling or predictive analysis
